# Faunistic diversity of spiders (Araneae) in Galichitsa mountain (FYR Macedonia)

**DOI:** 10.3897/BDJ.1.e977

**Published:** 2013-09-16

**Authors:** Christo Deltshev, Marjan Komnenov, Gergin Blagoev, Teodor Georgiev, Stoyan Lazarov, Emilija Stojkoska, Maria Naumova

**Affiliations:** † Institute of Biodiversity and Ecosystem Research, Bulgarian Academy of Sciences, Sofia, Bulgaria; ‡ Macedonian Museum of Natural History, Skopje, Macedonia; § Biodiversity Institute of Ontario, University of Guelph, Guelph, Canada; | Pensoft Publishers, Sofia, Bulgaria; ¶ National Museum of Natural History, Bulgarian Academy of Sciences, Sofia, Bulgaria

**Keywords:** Spiders, taxonomy, faunistic, zoogeography, Galichitsa Mt.

## Abstract

A total of 294 species from 31 families have been found in Galichitsa Mt. Of them, 85 species are new to the mountain, while 20 are also new to the fauna of FYR of Macedonia. According to their current distribution the established species can be assigned to 17 zoogeographical categories, grouped into 5 complexes (Cosmopolitan, Holarctic, European, Mediterranean, Endemics of Balkans). Dominant are Holarctic species (66.0%) followed by European (16.5%) and Mediterranean (9.3%). The endemics (6.2%) and Southeast European species (1.7%) emphasize the local character of this fauna, but its low percentage suggests an important process of colonization.

## Introduction

**Figure 1.**
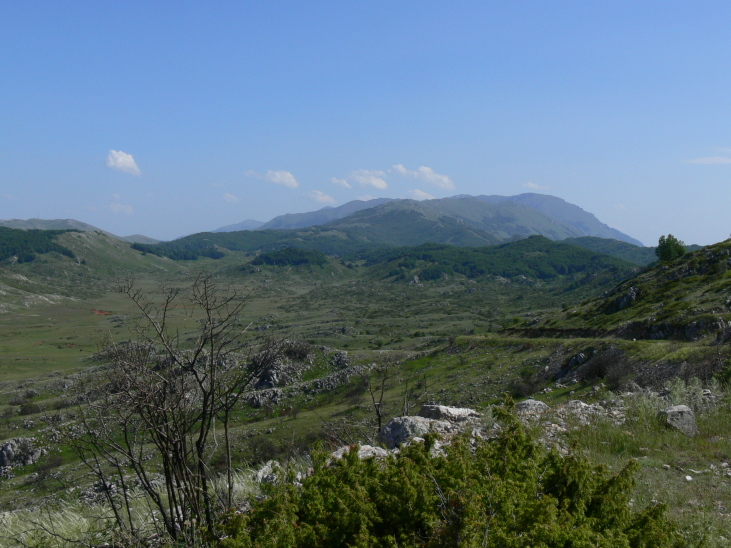
Galichitsa Mt, landscape view.

**Figure 2.**
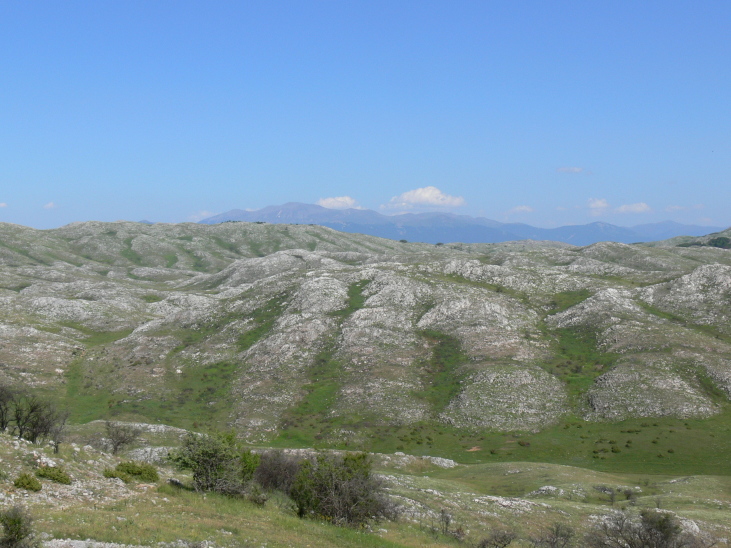
Galichitsa Mt, karst forms.

**Figure 3.**
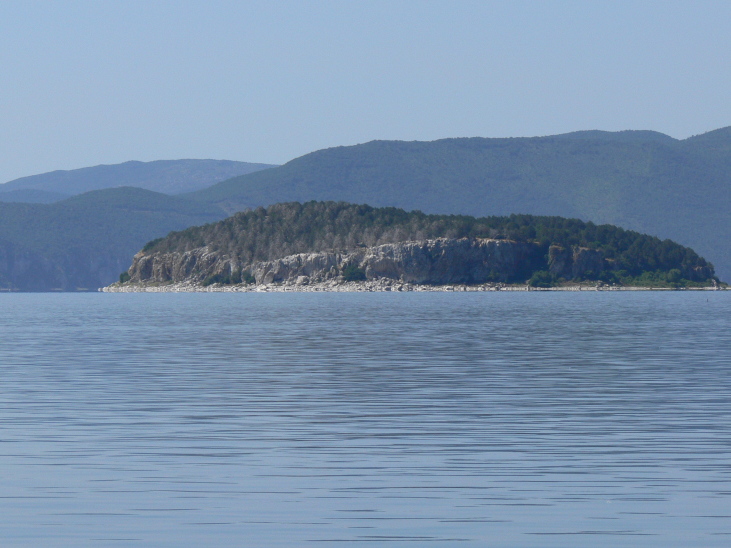
Golem Grad Island, a part of Galichitsa Mt.

Galichitsa Mt is situated between the lakes Ohrid and Prespa. It is a long, narrow mountain, extending in a North-South direction, continuing in Albania Southwardly. The side of the mountain facing Ohrid Lake is steep, rocky and to a great extent bare, thus resembling the mountains of the Dinaric Alps. This, and the presence of associated dry valleys, makes this part of Galichitsa difficult to access. The opposite, eastern side of Galichitsa, however, is quite different, with slant slopes completely overgrown with forests that go down to the Prespa Valley. Regarding its morphogenesis, the origin of Galichitsa is related to radial tectonic movements that took place in the Tertiary period, when this mountain massif actually developed. The geological structure is mainly limestone. Erosion of the karstic material has left deep traces in the rock, forming all kinds of relief: crevices, hollows, depressions and karstic fields. From a geological standpoint, the island “Golem Grad” situated in Lake Prespa, also belongs to the Galichitsa Mountain, which was split in the past by tectonic movements (Reed et al. 2004) (Figs 1, 2, 3).

The first reports for the spider fauna of Galichitsa Mt. came from Drensky (1929), Drensky (1936) and Stojićević (1929). This information was summarized later by Drensky (1936), who reported 83 species. Additional data can be found again in the papers of Šilhavý (1944), Nikolić and Polenec (1981), Deeleman-Reinhold and Deeleman (1988), Deltshev et al. (2000), Lazarov (2004), Wunderlich (1973) and Wunderlich (1985). All these data are summarized by Blagoev (2002). The aim of this study is to present a review of the spider fauna of Galichitsa Mt. due to critical incorporation of available literature data and unpublished faunistic records carried out by sporadic research in the last 10 years.

## Materials and methods

**Figure 4.**
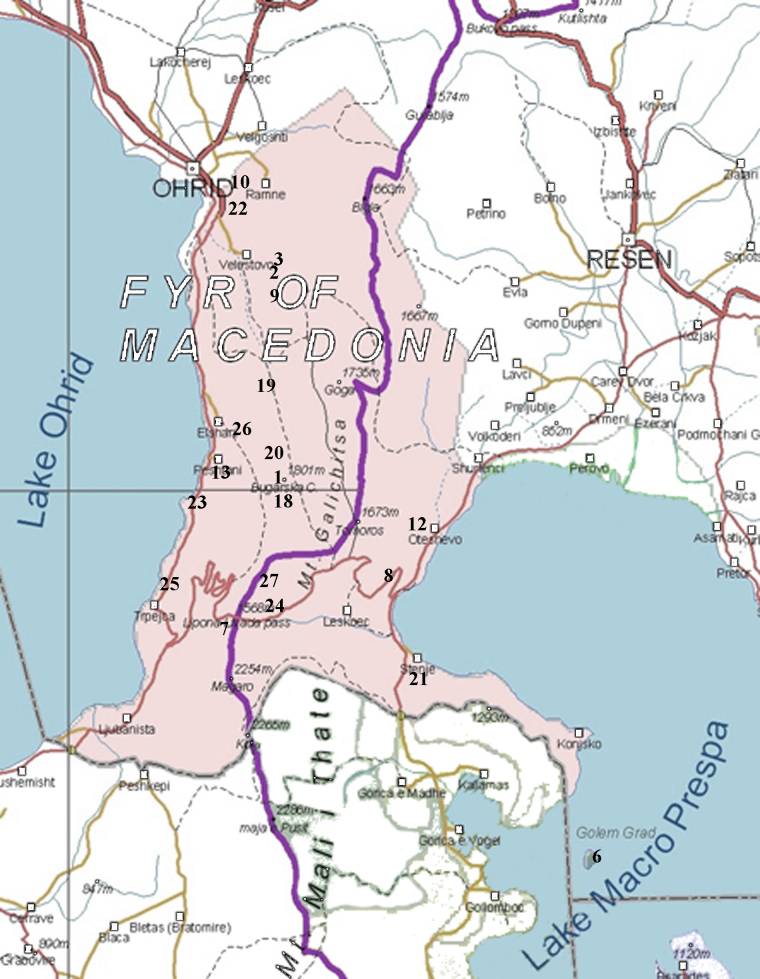
Map of the localities where the spiders have been collected in Galichitsa Mt.

**Figure 5.**
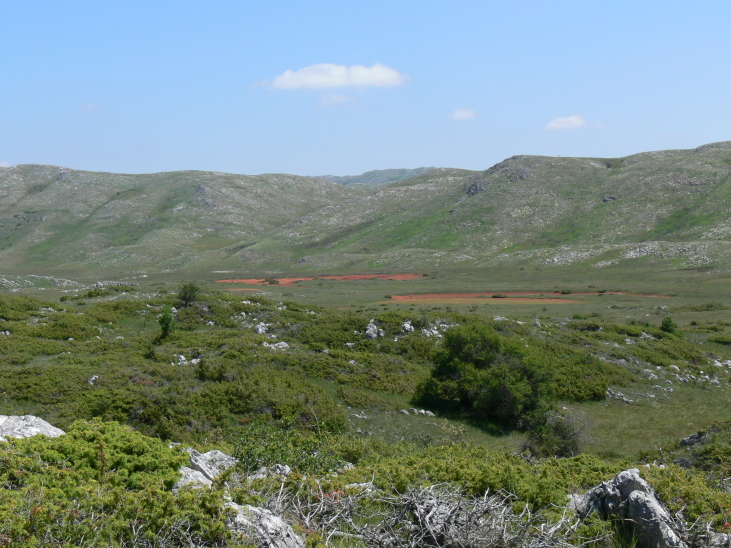
Sampling site at Crvena lokva.

**Figure 6.**
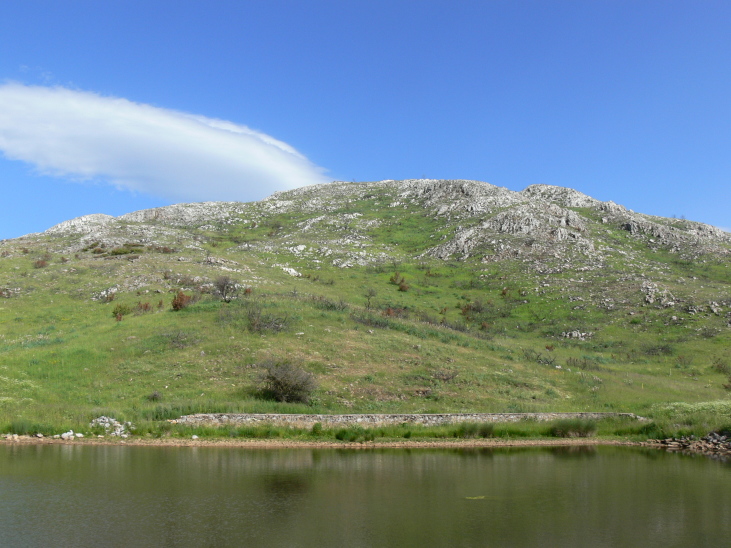
Sampling site at Dzhafa pool.

**Figure 7.**
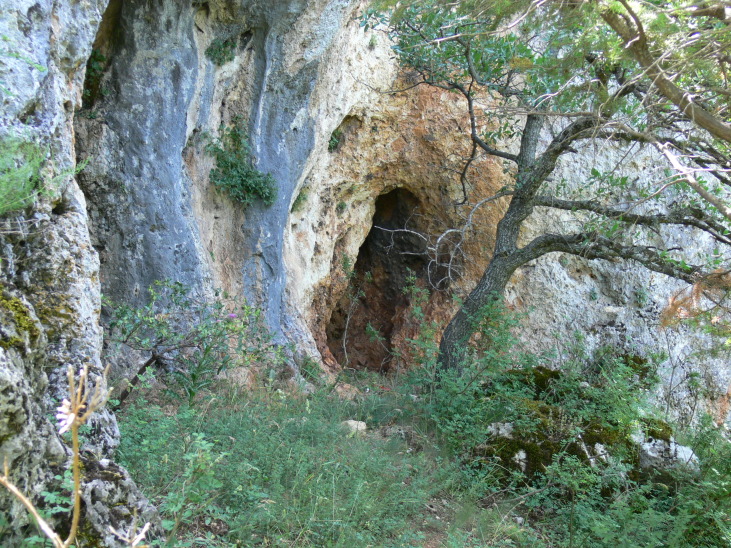
Sampling site at Leskovska Pestera Cave.

The spider material treated herein comes from two major sources. The first part comprises critical incorporation of all available literature records concerning the distribution of spiders of Galichitsa Mt. The second part comprises original collections made in the last 10 years during field surveys covering most of the territory of the mountain (Fig. 4, Table 1). The spiders have been collected mainly by hand, under stones, by sweeping and sewing (Figs 5, 6, 7). The names of the collectors: P. Beron (P.B.), G. Blagoev (G.B.), C. Deltshev (C.D.), M. Komnenov (M.K.), S. Lazarov (S.L.), B. Petrov (B.P.), E. Stojkoska (E.S.). and D. Vidincheva (D.V.) are mentioned by their abbreviations in Table 1. The taxonomic arrangements of the species list follow Platnick (2013). The material is deposited in the Natural History Museum (Skopje) and National Museum of Natural History (Sofia).

## Checklists

### Checklist of the spiders in Galichitsa Mts

#### ATYPIDAE

#### Atypus afinis

Eichwald, 1830

##### Distribution

West Palearctic.

##### Notes

Previously recorded from Resen and Ohrid (Drensky 1929, Drensky 1936).

#### SCYTODIDAE

#### Scytodes thoracica

(Latreille, 1802)

##### Materials

- **Type status:** Other material. **Occurrence:** recordedBy: C. Deltshev & E. Stojkoska; sex: 1 female; **Location:** country: FYR of Macedonia; locality: Galichitsa Mt., Peshtani vill.; verbatimElevation: 719 m; **Event:** eventDate: 31-08-2005
- **Type status:** Other material. **Occurrence:** recordedBy: C. Deltshev & M. Komnenov; sex: 1 female; **Location:** country: FYR of Macedonia; locality: Galichitsa Mt., Stenje vill., Stenjsko Blato bog; verbatimElevation: 850 m; **Event:** eventDate: 17-06-2008
- **Type status:** Other material. **Occurrence:** recordedBy: C. Deltshev & M. Komnenov; sex: 2 females; **Location:** country: FYR of Macedonia; locality: Galichitsa Mt., vill. Konjsko, Golem Grad island; verbatimElevation: 855 m; **Event:** eventDate: 20-06-2008

##### Distribution

Holarctic.

##### Notes

Previously recorded from Resen and Ohrid (Drensky 1929, Drensky 1936).

#### PHOLCIDAE

#### Holocnemus pluchei

(Scopoli, 1763)

##### Materials

- **Type status:** Other material. **Occurrence:** recordedBy: C. Deltshev & E. Stojkoska; sex: 1 female; **Location:** country: FYR of Macedonia; locality: Galichitsa Mt., Peshtani vill.; verbatimElevation: 719 m; **Event:** eventDate: 31-08-2005
- **Type status:** Other material. **Occurrence:** recordedBy: C. Deltshev & M. Komnenov; sex: 1 male, 1 female; **Location:** country: FYR of Macedonia; locality: Galichitsa Mt., Stenje vill., Stenjsko Blato bog; verbatimElevation: 850 m; **Event:** eventDate: 17-06-2008

##### Distribution

Mediterranean.

##### Notes

First record for NP Galitshitsa

#### Pholcus opilionoides

(Schrank, 1781)

##### Distribution

European.

##### Notes

Previously recorded from Resen and Ohrid (Drensky 1929, Drensky 1936).

#### Pholcus phalangioides

(Fuesslin, 1775)

##### Distribution

Cosmopolitan.

##### Notes

Previously recorded from unspecified locality between Resen and Ohrid (Drensky 1929, Drensky 1936).

#### Spermophora senoculata

(Duges, 1836)

##### Materials

- **Type status:** Other material. **Occurrence:** recordedBy: C. Deltshev & E. Stojkoska; sex: 1 female; **Location:** country: FYR of Macedonia; locality: Galichitsa Mt., Peshtani vill.; verbatimElevation: 719 m; **Event:** eventDate: 31-08-2005

##### Distribution

Holarctic.

##### Notes

Previously recorded from Resen and Ohrid (Drensky 1929, Drensky 1936).

#### SEGESTRIIDAE

#### Segestria bavarica

C.L. Koch, 1843

##### Materials

- **Type status:** Other material. **Occurrence:** recordedBy: C. Deltshev & G. Blagoev; sex: 1 male; **Location:** country: FYR of Macedonia; locality: Galichitsa Mt., Resen; verbatimElevation: 1000 m; **Event:** eventDate: 30-08-2002

##### Distribution

European.

##### Notes

First rcord in Galichitsa Mt.

#### Segestria senoculata

(Linnaeus, 1758)

##### Materials

- **Type status:** Other material. **Occurrence:** recordedBy: C. Deltshev & M. Komnenov; sex: 5 females; **Location:** country: FYR of Macedonia; locality: Galichitsa Mt., Tomoros peak; verbatimElevation: 1830 m; **Event:** eventDate: 22-06-2008

##### Distribution

Palearctic.

##### Notes

First record in Galichitsa Mt.

#### DYSDERIDAE

#### Dysdera erythrina

(Welckenaer, 1802)

##### Distribution

European.

##### Notes

Previously recorded from unspecified locality between Resen and Ohrid (Drensky 1929).

#### Dysdera pectinata

Deleman-Reinhold, 1988

##### Distribution

Balkan endemic.

##### Notes

Previously recorded from unspecified locality between Resen and Ohrid (Deeleman-Reinhold and Deeleman 1988)

#### Dysdera longirostris

Doblika, 1853

##### Distribution

East European.

##### Notes

Previously recorded from Galichitsa NP (Otechevo) (Deeleman-Reinhold and Deeleman 1988).

#### Dysderocrates storkani

(Kratochvil, 1935)

##### Distribution

Balkan endemic.

##### Notes

Previously recorded from Galichitsa NP (1100 – 1400) (Deeleman-Reinhold and Deeleman 1988).

#### Harpactea lepida

(C. L. Koch, 1838)

##### Distribution

European.

##### Notes

Previously recorded from unspecified locality between Resen and Ohrid (Drensky 1929, Drensky 1936).

#### MIMETIDAE

#### Ero cambridgei

Kulczyn’ski, 1911

##### Materials

- **Type status:** Other material. **Occurrence:** recordedBy: C. Deltshev & M. Komnenov; sex: 1 female; **Location:** country: FYR of Macedonia; locality: Galichitsa Mt., Stenje vill., Stenjsko Blato bog; verbatimElevation: 850 m; **Event:** eventDate: 18-06-2008

##### Distribution

Palearctic.

##### Notes

First record in FYR of Macedonia.

#### ULOBORIDAE

#### Uloborus walckenaerius

Latreille, 1806

##### Distribution

Palearctic.

##### Notes

Previously recorded from Ohrid (Drensky 1929, Drensky 1936).

#### NESTICIDAE

#### Nesticus cellulanus

(Clerck, 1757)

##### Materials

- **Type status:** Other material. **Occurrence:** recordedBy: C. Deltshev & M. Komnenov; sex: 2 males, 3 females; **Location:** country: FYR of Macedonia; locality: Galichitsa Mt., Leskovec vill., Leskovska Peshtera cave; verbatimElevation: 1066 m; **Event:** eventDate: 18-06-2008

##### Distribution

Holarctic.

##### Notes

First record in Galichitsa Mt.

#### THERIDIIDAE

#### Asagena phalerata

(Panzer, 1801)

##### Distribution

Palearctic.

##### Notes

Previously recorded from unspecified locality between Resen and Ohrid (Drensky 1929, Drensky 1936).

#### Crustulina guttata

(Wider, 1834)

##### Materials

- **Type status:** Other material. **Occurrence:** recordedBy: C. Deltshev & E. Stojkoska; sex: 2 females; **Location:** country: FYR of Macedonia; locality: Galichitsa Mt., Peshtani vill; verbatimElevation: 719 m; **Event:** eventDate: 31-08-2005

##### Distribution

Palearctic.

##### Notes

Previously recorded from unspecified locality between Ohrid and Resen (Drensky 1929, Drensky 1936).

#### Crustulina scabripes

Simon, 1881

##### Materials

- **Type status:** Other material. **Occurrence:** recordedBy: C. Deltshev & M. Komnenov; sex: 2 females, 1 juv.; **Location:** country: FYR of Macedonia; locality: Galichitsa Mt., Dzhafa pool; verbatimElevation: 1650 m; **Event:** eventDate: 18-06-2008

##### Distribution

Mediterranean.

##### Notes

First record in FYR of Macedonia.

#### Crustulina sticta

(O. P.-Cambridge, 1861)

##### Materials

- **Type status:** Other material. **Occurrence:** recordedBy: C. Deltshev & E. Stojkoska; sex: 1 female; **Location:** country: FYR of Macedonia; locality: Galichitsa Mt., Peshtani vill.; verbatimElevation: 719 m; **Event:** eventDate: 31-08-2005
- **Type status:** Other material. **Occurrence:** recordedBy: C. Deltshev & M. Komnenov; sex: 1 male; **Location:** country: FYR of Macedonia; locality: Galichitsa Mt., Prespa lake, village Konjsko, Golem Grad island; verbatimElevation: 842 m; **Event:** eventDate: 20-06-2008

##### Distribution

Holarctic.

##### Notes

First record in FYR of Macedonia.

#### Enoplognatha latimana

Hippa & Oksala, 1982

##### Materials

- **Type status:** Other material. **Location:** country: FYR of Macedonia; locality: Galichitsa Mt., Preseka; verbatimElevation: 1603 m; **Event:** eventDate: 30-08-2005

##### Distribution

Holarctic.

##### Notes

First record in Galichitsa Mt.

#### Enoplognatha thoracica

(Hahn, 1833)

##### Distribution

Holarctic.

##### Notes

Previously recorded from unspecified locality between Resen and Ohrid (Drensky 1929, Drensky 1936).

#### Episinus truncatus

Latreille, 1809

##### Distribution

Palearctic.

##### Notes

Previously recorded from unspecified locality between Resen and Ohrid (Drensky 1929, Drensky 1936).

#### Euryopis episinoides

(Walckenaer, 1847)

##### Distribution

Mediterranean.

##### Notes

Previously recorded from Ohrid (Drensky 1929).

#### Euryopis quinqueguttata

Thorell, 1875

##### Distribution

European.

##### Notes

Previously recorded from Ohrid (Stojićević 1929).

#### Heterotheridion nigrovariegatum

(Simon, 1873)

##### Distribution

Palearctic.

##### Notes

Previously recorded from Ohrid and uspecified locality between Resen and Ohrid (Drensky 1929, Drensky 1936).

#### Lasaeola tristis

(Hahn, 1833)

##### Materials

- **Type status:** Other material. **Occurrence:** recordedBy: C. Deltshev & M. Komnenov; sex: 1 male, 2 females; **Location:** country: FYR of Macedonia; locality: Galichitsa Mt., Simoncheska Lokva pool; verbatimElevation: 1680 m; **Event:** eventDate: 18-06-2008

##### Distribution

European.

##### Notes

First record in FYR of Macedonia.

#### Neottiura bimaculata

(Linnaeus, 1767)

##### Distribution

Holarctic.

##### Notes

Previously recorded from unspecified locality betweenResen - Ohrid (Drensky 1929, Drensky 1936).

#### Parasteatoda lunata

(Clerck, 1757)

##### Distribution

Palearctic.

##### Notes

Previously recorded from unspecified locality betweenResen and Ohrid (Drensky 1929, Drensky 1936).

#### Parasteatoda tepidariorum

(C. L. Koch, 1841)

##### Distribution

Cosmopolitan.

##### Notes

Previously recorded from unspecified locality between Resen and Ohrid (Drensky 1929, Drensky 1936).

#### Phylloneta impressa

(L. Koch, 1881)

##### Materials

- **Type status:** Other material. **Occurrence:** recordedBy: C. Deltshev & M. Komnenov; sex: 1 male; **Location:** country: FYR of Macedonia; locality: Galichitsa Mt., Crvena Lokva pool; verbatimElevation: 1620 m; **Event:** eventDate: 20-06-2008
- **Type status:** Other material. **Occurrence:** recordedBy: C. Deltshev & M. Komnenov; sex: 2 males; **Location:** country: FYR of Macedonia; locality: Galichitsa Mt., Simoncheska Lokva pool; verbatimElevation: 1680 m; **Event:** eventDate: 18-06-2008
- **Type status:** Other material. **Occurrence:** recordedBy: C. Deltshev & M. Komnenov; sex: 1 male; **Location:** country: FYR of Macedonia; locality: Galichitsa Mt., Stenje vill., Stenjsko Blato bog; verbatimElevation: 850 m; **Event:** eventDate: 17-06-2008

##### Distribution

Holarctic.

##### Notes

Previously recorded from unspecified locality between Resen and Ohrid (Drensky 1929, Drensky 1936).

#### Steatoda bipunctata

(Linnaeus, 1758)

##### Distribution

Holarctic.

##### Notes

Previously recorded from unspecified locality between Resen and Ohrid (Drensky 1929, Drensky 1936).

#### Phylloneta sisyphia

(Clerk, 1757)

##### Materials

- **Type status:** Other material. **Occurrence:** recordedBy: C. Deltshev & M. Komnenov; sex: 1 male; **Location:** country: FYR of Macedonia; locality: Galichitsa Mt., Simoncheska Lokva pool; verbatimElevation: 1680 m; **Event:** eventDate: 18-06-2008

##### Distribution

Palearctic.

##### Notes

First record in Galichitsa Mt.

#### Platnickina tincta

(Walckenaer, 1802)

##### Materials

- **Type status:** Other material. **Occurrence:** recordedBy: D. Vidincheva; **Location:** country: FYR of Macedonia; locality: Galichitsa Mt.; verbatimElevation: 600-1800 m; **Event:** eventDate: 26-10-1992

##### Distribution

Holarctic.

##### Notes

Previously recorded from unspecified locality between Resen and Ohrid (Drensky 1929).

#### Simitidion simile

(C. L. Koch, 1836)

##### Distribution

Holarctic.

##### Notes

Previously recorded from Ohrid (Drensky 1929, Drensky 1936).

#### Steatoda castanea

(Clerck, 1757)

##### Materials

- **Type status:** Other material. **Occurrence:** recordedBy: C. Deltshev & G. Blagoev; sex: 1 female; **Location:** country: FYR of Macedonia; locality: Galichitsa Mt., Ohrid, Studenchitsa; verbatimElevation: 690 m; **Event:** eventDate: 30-08-2002
- **Type status:** Other material. **Occurrence:** recordedBy: C. Deltshev & M. Komnenov; sex: 1 female; **Location:** country: FYR of Macedonia; locality: Galichitsa Mt., Stenje vill., Stenjsko Blato bog; verbatimElevation: 850 m; **Event:** eventDate: 17-06-2008

##### Distribution

Palearctic.

##### Notes

Previously recorded from unspecified locality between Resen and Ohrid (Drensky 1929, Drensky 1936).

#### Steatoda meridionalis

(Kulczyński, 1894)

##### Materials

- **Type status:** Other material. **Occurrence:** recordedBy: D. Vidincheva; **Location:** country: FYR of Macedonia; locality: Galichitsa Mt.; verbatimElevation: 600-1800 m; **Event:** eventDate: 26-10-1992

##### Distribution

East European.

##### Notes

First record in Galichitsa Mt.

#### Steatoda paykulliana

(Walckenaer, 1805)

##### Materials

- **Type status:** Other material. **Occurrence:** recordedBy: C. Deltshev & M. Komnenov; sex: 1 female; **Location:** country: FYR of Macedonia; locality: Galichitsa Mt., vill. Stenje, Stenjsko Blato bog; verbatimElevation: 850 m; **Event:** eventDate: 17-06-2008

##### Distribution

West Palearctic.

##### Notes

First record in Galichitsa Mt.

#### Steatoda triangulosa

(Walckenaer, 1802)

##### Materials

- **Type status:** Other material. **Occurrence:** recordedBy: C. Deltshev & E. Stojkoska; sex: 1 female; **Location:** country: FYR of Macedonia; locality: Galichitsa Mt., Peshtani vill.; verbatimElevation: 719 m; **Event:** eventDate: 31-08-2005
- **Type status:** Other material. **Occurrence:** recordedBy: C. Deltshev & M. Komnenov; sex: 2 females; **Location:** country: FYR of Macedonia; locality: Galichitsa Mt., Prespa lake, vill. Konjsko, Golem Grad island; verbatimElevation: 842 m; **Event:** eventDate: 20-06-2008

##### Distribution

Cosmopolitan.

##### Notes

Previously recorded from unspecified locality between Resen and Ohrid (Drensky 1929, Drensky 1936).

#### Theridion melanurum

Hahn, 1831

##### Distribution

Holarctic.

##### Notes

Previously recorded from Ohrid (Drensky 1929, Drensky 1936).

#### LINYPHIIDAE

#### Areoncus humilis

(Blackwall, 1841)

##### Distribution

Palearctic.

##### Notes

Previously recorded from Ohrid, Studenchitsa (Drensky 1929, Drensky 1936).

#### Centromerus acutidentatus

Deltshev, 2002

##### Materials

- **Type status:** Other material. **Occurrence:** recordedBy: C. Deltshev & M. Komnenov; sex: 1 female; **Location:** country: FYR of Macedonia; locality: Galichitsa Mt., Vojla cave; verbatimElevation: 1508 m; **Event:** eventDate: 20-06-2008

##### Distribution

Balkan endemic.

##### Notes

First record in Galichitsa Mt.

#### Centromerus sp.

##### Materials

- **Type status:** Other material. **Occurrence:** recordedBy: C. Deltshev & M. Komnenov; sex: 1 female; **Location:** country: FYR of Macedonia; locality: Galichitsa Mt., Vojla cave; verbatimElevation: 1508 m; **Event:** eventDate: 20-06-2008

##### Notes

First record in Galichitsa Mt.

#### Crosbyarachne silvestris

(Georgescu, 1973)

##### Materials

- **Type status:** Other material. **Occurrence:** recordedBy: D. Vidincheva; **Location:** country: FYR of Macedonia; locality: Galichitsa Mt.; verbatimElevation: 600-1800 m; **Event:** eventDate: 26-10-1992

##### Distribution

Middle and Southeast European.

##### Notes

First record in FYR of Macedonia.

#### Diplocephalus cristatus

(Blackwall, 1833)

##### Materials

- **Type status:** Other material. **Occurrence:** recordedBy: C. Deltshev & M. Komnenov; sex: 1 male; **Location:** country: FYR of Macedonia; locality: Galichitsa Mt., Zhichara; verbatimElevation: 1515 m; **Event:** eventDate: 20-06-2008

##### Distribution

Holarctic.

##### Notes

First record in Galichitsa Mt.

#### Erigone dentipalpis

(Wider, 1834)

##### Distribution

Holarctic.

##### Notes

Previously recorded from Ohrid, Studenchitsa (Drensky 1929, Drensky 1936).

#### Frontinellina frutetorum

(C. L. Koch, 1834)

##### Materials

- **Type status:** Other material. **Occurrence:** recordedBy: C. Deltshev & M. Komnenov; sex: 1 female; **Location:** country: FYR of Macedonia; locality: Galichitsa Mt., Crvena Lokva pool; verbatimElevation: 1620 m; **Event:** eventDate: 20-06-2008
- **Type status:** Other material. **Occurrence:** recordedBy: C. Deltshev & G. Blagoev; sex: 1 male; **Location:** country: FYR of Macedonia; locality: Galichitsa Mt., Ohrid, Studenchitsa; verbatimElevation: 690 m; **Event:** eventDate: 30-08-2002
- **Type status:** Other material. **Occurrence:** recordedBy: C. Deltshev & M. Komnenov; sex: 4 females; **Location:** country: FYR of Macedonia; locality: Galichitsa Mt., Simoncheska Lokva pool; verbatimElevation: 1680 m; **Event:** eventDate: 18-06-2008

##### Distribution

Palearctic.

##### Notes

First record in Galichitsa Mt.

#### Gongilidium rufipes

(Linnaeus, 1758)

##### Distribution

Palearctic.

##### Notes

Previously recorded from unspecified locality between Resen and Ohrid (Drensky 1929, Drensky 1936).

#### Incestophantes crucifer

(Menge, 1866)

##### Distribution

Palearctic.

##### Notes

Previously recorded from Ohrid (Stojićević 1929).

#### Improphantes decolor

(Westring, 1861)

##### Materials

- **Type status:** Other material. **Occurrence:** recordedBy: C. Deltshev & M. Komnenov; sex: 1 male; **Location:** country: FYR of Macedonia; locality: Galichitsa Mt., Zhichara; verbatimElevation: 1515 m; **Event:** eventDate: 20-06-2008

##### Distribution

West Palearctic.

##### Notes

First record for FYR of Macedonia.

#### Ipa keyserlingi

(Ausserer, 1867)

##### Distribution

Palearctic.

##### Notes

Previously recorded from unspecified locality between Resen and Ohrid (Drensky 1929, Drensky 1936).

#### Lepthyphantes centromeroides

Kulczyński, 1914

##### Materials

- **Type status:** Other material. **Occurrence:** recordedBy: C. Deltshev & M. Komnenov; sex: 1 female; **Location:** country: FYR of Macedonia; locality: Galichitsa Mt., Vojla cave; verbatimElevation: 1508 m; **Event:** eventDate: 20-06-2008

##### Distribution

Balkan endemic.

##### Notes

First record in Galichitsa Mt.

#### Lephthyphantes leprosus

(Ohlert, 1865)

##### Materials

- **Type status:** Other material. **Occurrence:** recordedBy: C. Deltshev & M. Komnenov; sex: 1 male, 1 female; **Location:** country: FYR of Macedonia; locality: Galichitsa Mt., Leskovec vill., Leskovska Peshtera cave; verbatimElevation: 1066 m; **Event:** eventDate: 18-06-2008

##### Distribution

Holarctic.

##### Notes

Previously recorded from unspecified locality between Resen and Ohrid (Drensky 1929, Drensky 1936).

#### Linyphia hortensis

Sundevall, 1830

##### Materials

- **Type status:** Other material. **Occurrence:** recordedBy: D. Vidincheva; **Location:** country: FYR of Macedonia; locality: Galichitsa Mt.; verbatimElevation: 600-1800 m; **Event:** eventDate: 26-10-1992

##### Distribution

Palearctic.

##### Notes

Previously recorded from unspecified locality between Ohrid and Resen (Drensky 1929, Drensky 1936, Stojićević 1929).

#### Linyphia triangularis

(Clerck, 1757)

##### Materials

- **Type status:** Other material. **Occurrence:** recordedBy: C. Deltshev & G. Blagoev; sex: 1 male; **Location:** country: FYR of Macedonia; locality: Galichitsa Mt., Ohrid, Studenchitsa; verbatimElevation: 690 m; **Event:** eventDate: 30-08-2002
- **Type status:** Other material. **Occurrence:** recordedBy: C. Deltshev & E. Stojkoska; sex: 1 male, 2 females; **Location:** country: FYR of Macedonia; locality: Galichitsa Mt., Stenje vill., Stenjsko Blato bog; verbatimElevation: 850 m; **Event:** eventDate: 30-08-2005

##### Distribution

Palearctic.

##### Notes

Previously recorded from unspecified locality between Ohrid and Resen (Drensky 1929, Drensky 1936).

#### Micrargus herbigradus

(Blackwall, 1854)

##### Materials

- **Type status:** Other material. **Occurrence:** recordedBy: C. Deltshev & M. Komnenov; sex: 1 male; **Location:** country: FYR of Macedonia; locality: Galichitsa Mt., road to Bugarska Chuka peak; verbatimElevation: 1509 m; **Event:** eventDate: 20-06-2008

##### Distribution

Palearctic.

##### Notes

First record in FYR of Macedonia.

#### Microneta viaria

(Blackwall, 1841)

##### Materials

- **Type status:** Other material. **Occurrence:** recordedBy: C. Deltshev & G. Blagoev; sex: 1 male; **Location:** country: FYR of Macedonia; locality: Galichitsa Mt., Resen; verbatimElevation: 1000 m; **Event:** eventDate: 30-08-2002

##### Distribution

Holarctic.

#### Meioneta rurestris

(C. L. Koch, 1836)

##### Distribution

Palearctic.

##### Notes

Previously recorded from unspecified locality between Resen and Ohrid (Drensky 1929, Drensky 1936).

#### Megalepthyphantes collinus

(L. Koch, 1892)

##### Materials

- **Type status:** Other material. **Occurrence:** recordedBy: C. Deltshev & E. Stojkoska; sex: 2 females; **Location:** country: FYR of Macedonia; locality: Galichitsa Mt., vill. Stenje, Stenjsko Blato bog; verbatimElevation: 850 m; **Event:** eventDate: 30-08-2005

##### Distribution

Palearctic.

##### Notes

First record in Galichitsa Mt.

#### Megalepthyphantes nebulosus

(Sundevall, 1830)

##### Materials

- **Type status:** Other material. **Occurrence:** recordedBy: C. Deltshev; sex: 2 female; **Location:** country: FYR of Macedonia; locality: Galichitsa Mt., Ohrid, Studenchitsa; verbatimElevation: 690 m; **Event:** eventDate: 01-09-2005

##### Distribution

Holarctic.

##### Notes

First record in Galichitsa Mt.

#### Microlinyphia pusilla

(Sundevall, 1830)

##### Distribution

Holarctic.

##### Notes

Previously recorded from Ohrid (Drensky 1929, Drensky 1936).

#### Microneta viaria

(Blackwall, 1841)

##### Materials

- **Type status:** Other material. **Occurrence:** recordedBy: C. Deltshev & M. Komnenov; sex: 1 female; **Location:** country: FYR of Macedonia; locality: Galichitsa Mt., road to Bugarska Chuka peak; verbatimElevation: 1509 m; **Event:** eventDate: 20-06-2008

##### Distribution

Holarctic.

##### Notes

First record in Galichitsa Mt.

#### Neriene clathrata

(Sundeval, 1830)

##### Materials

- **Type status:** Other material. **Occurrence:** recordedBy: C. Deltshev & M. Komnenov; sex: 1 female; **Location:** country: FYR of Macedonia; locality: Galichitsa Mt., Stenje vill., Stenjsko Blato bog; verbatimElevation: 850 m; **Event:** eventDate: 18-06-2008

##### Distribution

Holarctic.

##### Notes

Previously recorded from unspecified locality between Resen and Ohrid (Drensky 1929, Drensky 1936).

#### Neriene montana

(Clerck, 1757)

##### Distribution

Holarctic.

##### Notes

Previously recorded from Ohrid (Stojićević, 1929).

#### Neriene peltata

(Wider, 1854)

##### Distribution

Palearctic.

##### Notes

Previously recorded from unspecified locality between Resen and Ohrid (Drensky 1929, Drensky 1936).

#### Notioscopus sarcinatus

(O. P.-Cambridge, 1872)

##### Materials

- **Type status:** Other material. **Occurrence:** recordedBy: D. Vidincheva; **Location:** country: FYR of Macedonia; locality: Galichitsa Mt.; verbatimElevation: 600-1800 m; **Event:** eventDate: 26-10-1992

##### Distribution

European.

##### Notes

First record in FYR of Macedonia.

#### Oedothorax fuscus

(Blackwall, 1841)

##### Distribution

West Palearctic.

##### Notes

Previously recorded from Ohrid (Drensky 1929, Drensky 1936).

#### Palliduphantes byzantinus

(Fage, 1931)

##### Materials

- **Type status:** Other material. **Occurrence:** recordedBy: C. Deltshev & M. Komnenov; sex: 1 male, 2 females; **Location:** country: FYR of Macedonia; locality: Galichitsa Mt., Ohrid, Mechkina Dupka cave; verbatimElevation: 1020 m; **Event:** eventDate: 20-06-2008

##### Distribution

Balkan endemic.

##### Notes

First record in Galichitsa Mt.

#### Palliduphantes spelaeorum

(Kulczyński, 1914)

##### Distribution

Balkan endemic.

##### Notes

Previously recorded from Ohrid, Studenchitsa (Drensky 1929, Drensky 1936).

#### Palliduphantes trnovensis

(Drensky, 1931)

##### Materials

- **Type status:** Other material. **Occurrence:** recordedBy: C. Deltshev & M. Komnenov; sex: 2 males, 1 female, 2 juv.; **Location:** country: FYR of Macedonia; locality: Galichitsa Mt., Leskovec vill., Leskovska Peshtera cave; verbatimElevation: 1066 m; **Event:** eventDate: 18-06-2008

##### Distribution

Balkan endemic.

##### Notes

First record in Galichitsa Mt.

#### Pocadicnemis juncea

Locket & Millidge, 1953

##### Materials

- **Type status:** Other material. **Occurrence:** recordedBy: C. Deltshev & M. Komnenov; sex: 1 male; **Location:** country: FYR of Macedonia; locality: Galichitsa Mt., Stenje vill., Stenjsko Blato bog; verbatimElevation: 850 m; **Event:** eventDate: 18-06-2008

##### Distribution

Palearctic.

##### Notes

First record in FYR of Macedonia.

#### Porrhomma pygmaeum

(Blackwall, 1834)

##### Distribution

Palearctic.

##### Notes

Previously recorded from unspecified locality between Resen and Ohrid (Drensky 1929, Drensky 1936).

#### Prinerigone vagans

(Audouin, 1826)

##### Distribution

Subcosmopolitan.

##### Notes

Previously recorded from unspecified locality between Resen and Ohrid (Drensky 1929, Drensky 1936).

#### Tallusia experta

(O.P.-Cambridge, 1871)

##### Materials

- **Type status:** Other material. **Occurrence:** recordedBy: D. Vidincheva; **Location:** country: FYR of Macedonia; locality: Galichitsa Mt.; verbatimElevation: 600-1800 m; **Event:** eventDate: 26-10-1992

##### Distribution

Palearctic.

##### Notes

First record in Galichitsa Mt.

#### Tapinocyba biscissa

(O. P.-Cambridge, 1872)

##### Materials

- **Type status:** Other material. **Occurrence:** recordedBy: C. Deltshev & M. Komnenov; sex: 1 female; **Location:** country: FYR of Macedonia; locality: Galichitsa Mt., road to Bugarska Chuka peak; verbatimElevation: 1509 m; **Event:** eventDate: 20-06-2008

##### Distribution

Palearctic.

##### Notes

First record in FYR of Macedonia.

#### Tenuiphantes floriana

(van Helsdingen, 1977)

##### Materials

- **Type status:** Other material. **Occurrence:** recordedBy: D. Vidincheva; **Location:** country: FYR of Macedonia; locality: Galichitsa Mt.; verbatimElevation: 600-1800 m; **Event:** eventDate: 26-10-1992

##### Distribution

Southeast European.

##### Notes

First record in Galichitsa Mt.

#### Tenuiphantes tenuis

(Blackwall, 1852)

##### Materials

- **Type status:** Other material. **Occurrence:** recordedBy: D. Vidincheva; **Location:** country: FYR of Macedonia; locality: Galichitsa Mt.; verbatimElevation: 600-1800 m; **Event:** eventDate: 26-10-1992
- **Type status:** Other material. **Occurrence:** recordedBy: C. Deltshev & M. Komnenov; sex: 1 female; **Location:** country: FYR of Macedonia; locality: Galichitsa Mt., vill. Stenje, Stenjsko Blato bog; verbatimElevation: 850 m; **Event:** eventDate: 18-06-2008

##### Distribution

West Palearctic.

##### Notes

First record in Galichitsa Mt.

#### Trichoncus affinis

Kulczyn’ski, 1894

##### Distribution

Palearctic.

##### Notes

Previously recorded from Ohrid (Deltshev et al. 2000, Drensky 1929).

#### Troglohyphantes dalmaticus

(Kulczyn’ski, 1914)

##### Distribution

Balkan endemic.

##### Notes

Previously recorded from Ohrid, Studenchitsa (Drensky 1929, Drensky 1936).

#### Walckenaeria nudipalpis

(Westring, 1851)

##### Materials

- **Type status:** Other material. **Occurrence:** recordedBy: D. Vidincheva; **Location:** country: FYR of Macedonia; locality: Galichitsa Mt.; verbatimElevation: 600-1800 m; **Event:** eventDate: 26-10-1992

##### Distribution

Palearctic.

##### Notes

First record in Galichitsa Mt.

#### TETRAGNATHIDAE

#### Meta menardi

(Latreille, 1804)

##### Materials

- **Type status:** Other material. **Occurrence:** recordedBy: C. Deltshev & M. Komnenov; sex: 2 females; **Location:** country: FYR of Macedonia; locality: Galichitsa Mt., Vojla cave; verbatimElevation: 1508 m; **Event:** eventDate: 20-06-2008
- **Type status:** Other material. **Occurrence:** recordedBy: C. Deltshev & M. Komnenov; sex: 1 female, 1 juv.; **Location:** country: FYR of Macedonia; locality: Galichitsa Mt., Samatska Peshtera cave; verbatimElevation: 1436 m; **Event:** eventDate: 20-06-2008

##### Distribution

European.

##### Notes

**Figure 8.**
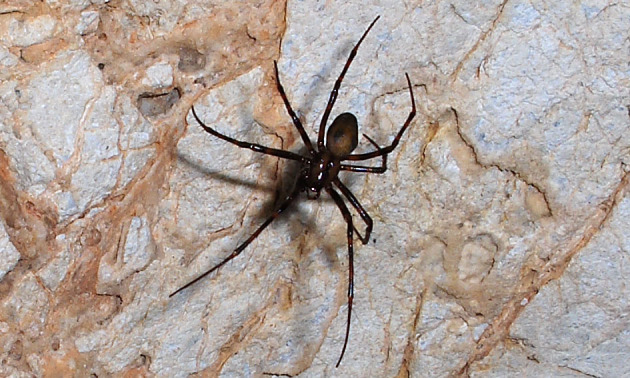
*Meta menardi*, a characteristic cave dweller.

First record in Galichitsa Mt. (Fig. 8).

#### Metellina mengei

(Blackwall, 1869)

##### Distribution

European.

##### Notes

Previously recorded from unspecified locality between Ohrid and Resen (Drensky 1929, Drensky 1936).

#### Metellina merianae

(Scopoli, 1763)

##### Distribution

European.

##### Notes

Previously recorded from Ohrid (Drensky 1929, Drensky 1936).

#### Mettelina segmentata

(Clerck, 1757)

##### Materials

- **Type status:** Other material. **Occurrence:** recordedBy: C. Deltshev; sex: 1 female; **Location:** country: FYR of Macedonia; locality: Galichitsa Mt., Ohrid, Studenchitsa; verbatimElevation: 690 m; **Event:** eventDate: 01-08-2005

##### Distribution

Palearctic.

##### Notes

Previously recorded from unspecified locality between Resen and Ohrid (Drensky 1929, Drensky 1936).

#### Pachygnatha clercki

Sundevall, 1823

##### Distribution

Holarctic.

##### Notes

Previously recorded from unspecified locality between Resen and Ohrid (Drensky 1929, Drensky 1936).

#### Pachygnatha clerckoides

Wunderlich, 1985

##### Distribution

Balkan endemic.

##### Notes

Previously recorded from Ohrid (Wunderlich 1985).

#### Pachygnatha degeeri

Sundevall, 1830

##### Distribution

Palearctic.

##### Notes

Previously recorded from unspecified locality between Ohrid and Resen (Drensky 1929, Drensky 1936).

#### Tetragnatha extensa

(Linnaeus, 1758)

##### Materials

- **Type status:** Other material. **Occurrence:** recordedBy: C. Deltshev & M. Komnenov; sex: 1 female; **Location:** country: FYR of Macedonia; locality: Galichitsa Mt., Stenje vill., Stenjsko Blato bog; verbatimElevation: 850 m; **Event:** eventDate: 18-06-2008

##### Distribution

Holarctic.

##### Notes

First record in Galichitsa Mt.

#### Tetragnatha montana

Simon, 1874

##### Materials

- **Type status:** Other material. **Occurrence:** recordedBy: C. Deltshev & M. Komnenov; sex: 1 female; **Location:** country: FYR of Macedonia; locality: Galichitsa Mt., Stenje vill., Stenjsko Blato bog; verbatimElevation: 850 m; **Event:** eventDate: 17-06-2008

##### Distribution

Palearctic.

##### Notes

Previously recorded from Ohrid (Drensky 1929, Drensky 1936).

#### Tetragnatha nitens

(Audouin, 1826)

##### Distribution

Cosmopolitan.

##### Notes

Previously recorded from Ohrid (Drensky 1929, Drensky 1936).

#### Tetragnatha obtusa

C. L. Koch, 1837

##### Materials

- **Type status:** Other material. **Occurrence:** recordedBy: C. Deltshev & M. Komnenov; sex: 1 female; **Location:** country: FYR of Macedonia; locality: Galichitsa Mt., Stenje vill., Stenjsko Blato bog; verbatimElevation: 850 m; **Event:** eventDate: 17-06-2008

##### Distribution

Palearctic.

##### Notes

First record in Galichitsa Mt.

#### Tetragnatha pinicola

L. Koch, 1870

##### Materials

- **Type status:** Other material. **Occurrence:** recordedBy: C. Deltshev & M. Komnenov; sex: 2 females; **Location:** country: FYR of Macedonia; locality: Galichitsa Mt., Tomoros peak; verbatimElevation: 1830 m; **Event:** eventDate: 22-06-2008

##### Distribution

Palearctic.

##### Notes

First record in FYR of Macedonia.

#### ARANEIDAE

#### Agalenatea redii

(Scopoli, 1763)

##### Materials

- **Type status:** Other material. **Occurrence:** recordedBy: C. Deltshev; sex: 1 female; **Location:** country: FYR of Macedonia; locality: Galichitsa Mt., Ohrid, Studenchitsa; verbatimElevation: 690 m; **Event:** eventDate: 01-08-2005

##### Distribution

Palearctic.

##### Notes

Unspecified locality between Ohrid - Resen (Drensky 1929, Drensky 1936, Stojićević 1929).

#### Aculepeira ceropegia

(Walckenaer, 1802)

##### Materials

- **Type status:** Other material. **Occurrence:** recordedBy: C. Deltshev & M. Komnenov; sex: 1 female; **Location:** country: FYR of Macedonia; locality: Galichitsa Mt., Tomoros peak; verbatimElevation: 1830 m; **Event:** eventDate: 22-06-2008

##### Distribution

Palearctic.

##### Notes

First record in Galichitsa Mt.

#### Araneus angulatus

Clerck, 1757

##### Distribution

Palearctic.

##### Notes

Previously recorded from unspecified locality between Resen and Ohrid (Drensky 1929, Drensky 1936).

#### Araneus circe

(Audouin, 1826)

##### Distribution

Palearctic.

##### Notes

Previously recorded from unspecified locality between Resen and Ohrid (Drensky 1929, Drensky 1936).

#### Araneus diadematus

Clerck, 1757

##### Materials

- **Type status:** Other material. **Occurrence:** recordedBy: C. Deltshev & G. Blagoev; sex: 1 male; **Location:** country: FYR of Macedonia; locality: Galichitsa Mt., Ohrid, Studenchitsa; verbatimElevation: 690 m; **Event:** eventDate: 30-08-2002

##### Distribution

Holarctic.

##### Notes

Previously recorded from Ohrid (Stojićević 1929).

#### Araneus grossus

(C. L. Koch, 1844)

##### Distribution

Europeo-Central Asiatic.

##### Notes

Previously recorded from Ohrid (Drensky 1929, Drensky 1936).

#### Araneus marmoreus

Clerck, 1757

##### Distribution

Holarctic.

##### Notes

Previously recorded from unspecified locality between Resen and Ohrid (Drensky 1929, Drensky 1936).

#### Araneus quadratus

Clerck, 1757

##### Distribution

Palearctic.

##### Notes

Previously recorded from Ohrid (Drensky 1929, Drensky 1936).

#### Araneus triguttatus

(Fabricius, 1793)

##### Distribution

Palearctic.

##### Notes

Previously recorded from unspecified locality between Resen and Ohrid (Drensky 1929, Drensky 1936).

#### Araniella cucurbitina

(Clerck, 1757)

##### Materials

- **Type status:** Other material. **Occurrence:** recordedBy: C. Deltshev & M. Komnenov; sex: 1 female; **Location:** country: FYR of Macedonia; locality: Galichitsa Mt., Stenje vill., Stenjsko Blato bog; verbatimElevation: 850 m; **Event:** eventDate: 17-06-2008

##### Distribution

Palearctic.

##### Notes

First record in Galichitsa Mt.

#### Argiope bruennichi

(Scopoli, 1772)

##### Distribution

Palearctic.

##### Notes

Previously recorded from Ohrid (Drensky 1929, Drensky 1936).

#### Cyclosa conica

(Pallas, 1772)

##### Materials

- **Type status:** Other material. **Occurrence:** recordedBy: C. Deltshev & M. Komnenov; sex: 1 female; **Location:** country: FYR of Macedonia; locality: Galichitsa Mt., Dzhafa pool; verbatimElevation: 1650 m; **Event:** eventDate: 18-06-2008

##### Distribution

Holarctic.

##### Notes

Previously recorded from Ohrid (Stojićević 1929).

#### Gibbaranea bituberculata

(Walck,, 1802)

##### Materials

- **Type status:** Other material. **Occurrence:** recordedBy: C. Deltshev & M. Komnenov; sex: 1 female; **Location:** country: FYR of Macedonia; locality: Galichitsa Mt., Leskovec, Leskovska Peshtera cave; verbatimElevation: 1066 m; **Event:** eventDate: 18-06-2008

##### Distribution

Palearctic.

##### Notes

First record in Galichitsa Mt.

#### Gibbaranea omoeda

(Thorell, 1870)

##### Distribution

Palearctic.

##### Notes

Previously recorded from Ohrid (Stojićević 1929).

#### Hypsosinga heri

(Hahn, 1831)

##### Materials

- **Type status:** Other material. **Occurrence:** recordedBy: C. Deltshev & M. Komnenov; sex: 1 female; **Location:** country: FYR of Macedonia; locality: Galichitsa Mt., Stenje vill., Stenjsko Blato bog; verbatimElevation: 850 m; **Event:** eventDate: 18-06-2008

##### Distribution

Palearctic.

##### Notes

First record in FYR of Macedonia.

#### Hipsosinga pygmaea

(Sundevall, 1831)

##### Distribution

Palearctic.

##### Notes

Previously recorded from Ohrid (Drensky 1929, Drensky 1936).

#### Larinioides ixobolus

(Thorell, 1873)

##### Distribution

Palearctic.

##### Notes

Previously recorded from unspecified locality between Ohrid - Resen (Drensky 1929, Drensky 1936).

#### Larinioides patagiatus

(Clerck, 1757)

##### Distribution

Holarctic.

##### Notes

Previously recorded from unspecified locality between Resen and Ohrid (Drensky 1929).

#### Larrinioides cornutus

(Clerck, 1757)

##### Distribution

Holarctic.

##### Notes

Previously recorded from Ohrid (Stojićević 1929).

#### Larrinioides sclopetarius

(Clerck, 1757)

##### Materials

- **Type status:** Other material. **Occurrence:** recordedBy: C. Deltshev & M. Komnenov; sex: 1 female; **Location:** country: FYR of Macedonia; locality: Galichitsa Mt., Tomoros peak; verbatimElevation: 01-03-05; **Event:** eventDate: 22-06-2008

##### Distribution

Holarctic.

##### Notes

Previously recorded from unspecified locality between Ohrid and Resen (Drensky 1929, Drensky 1936).

#### Larinioides suspicax

(O. P. Cambridge, 1876)

##### Materials

- **Type status:** Other material. **Occurrence:** recordedBy: C. Deltshev & M. Komnenov; sex: 1 female; **Location:** country: FYR of Macedonia; locality: Galichitsa Mt., Stenje vill., Stenjsko Blato bog; verbatimElevation: 850 m; **Event:** eventDate: 17-06-2008

##### Distribution

Europeo-Central Asiatic.

##### Notes

First record in Galichitsa Mt.

#### Mangora acalypha

(Walckenaer, 1802)

##### Materials

- **Type status:** Other material. **Occurrence:** recordedBy: C. Deltshev & M. Komnenov; sex: 1 male; **Location:** country: FYR of Macedonia; locality: Galichitsa Mt., Dzhafa pool; verbatimElevation: 1650 m; **Event:** eventDate: 18-06-2008
- **Type status:** Other material. **Occurrence:** recordedBy: C. Deltshev & M. Komnenov; sex: 2 females; **Location:** country: FYR of Macedonia; locality: Galichitsa Mt., Tomoros peak; verbatimElevation: 1830 m; **Event:** eventDate: 22-06-2008

##### Distribution

Palearctic.

##### Notes

Previously recorded from Ohrid (Stojićević 1929).

#### Neoscona adianta

(Walckenaer, 1802)

##### Materials

- **Type status:** Other material. **Occurrence:** recordedBy: C. Deltshev & M. Komnenov; sex: 1 male; **Location:** country: FYR of Macedonia; locality: Galichitsa Mt., Stenje vill., Stenjsko Blato bog; verbatimElevation: 850 m; **Event:** eventDate: 17-06-2008

##### Distribution

Palearctic.

##### Notes

Previously recorded from Ohrid and unspecified locality between Resen and Ohrid (Drensky 1929, Drensky 1936, Stojićević 1929).

#### Nuctenea silvicultrix

(C.L.Koch, 1835)

##### Materials

- **Type status:** Other material. **Occurrence:** recordedBy: C. Deltshev; sex: 1 female; **Location:** country: FYR of Macedonia; locality: Galichitsa Mt., Ohrid; verbatimElevation: 748 m; **Event:** eventDate: 31-08-2005

##### Distribution

Palearctic.

#### Nuctenea umbratica

Clerck, 1757

##### Materials

- **Type status:** Other material. **Occurrence:** recordedBy: C. Deltshev & M. Komnenov; sex: 1 male, 3 females; **Location:** country: FYR of Macedonia; locality: Galichitsa Mt., Stenje vill., Stenjsko Blato bog; verbatimElevation: 850 m; **Event:** eventDate: 17-06-2008

##### Distribution

European.

##### Notes

Previously recorded from unspecified locality between Resen and Ohrid (Drensky 1929, Drensky 1936).

#### Singa nitidula

C. L. Koch, 1844

##### Distribution

Palearctic.

##### Notes

Previously recorded from Ohrid (Drensky 1929, Drensky 1936).

#### Zilla diodia

(Walckenaer, 1802)

##### Distribution

European.

##### Notes

Previously recorded from Ohrid (Stojićević 1929).

#### LYCOSIDAE

#### Alopecosa accentuata

(Latreille, 1817)

##### Materials

- **Type status:** Other material. **Occurrence:** recordedBy: C. Deltshev & M. Komnenov; sex: 1 male; **Location:** country: FYR of Macedonia; locality: Galichitsa Mt., Stenje vill., Stenjsko Blato bog; verbatimElevation: 850 m; **Event:** eventDate: 17-06-2008

##### Distribution

Palearctic.

##### Notes

Previously recorded from Ohrid (Drensky 1929).

#### Alopecosa aculeata

(Clerck, 1757)

##### Materials

- **Type status:** Other material. **Occurrence:** recordedBy: C. Deltshev & M. Komnenov; sex: 1 male; **Location:** country: FYR of Macedonia; locality: Galichitsa Mt., Simoncheska Lokva pool; verbatimElevation: 1680 m; **Event:** eventDate: 18-06-2008

##### Distribution

Holarctic.

##### Notes

First record in Galichitsa Mt.

#### Alopecosa albofasciata

(Brullé, 1832)

##### Materials

- **Type status:** Other material. **Occurrence:** recordedBy: C. Deltshev & M. Komnenov; sex: 1 male; **Location:** country: FYR of Macedonia; locality: Galichitsa Mt., Dzhafa pool; verbatimElevation: 1650 m; **Event:** eventDate: 18-06-2008

##### Distribution

Mediterranean.

##### Notes

Previously recorded from Ohrid (Drensky 1929).

#### Alopecosa cuneata

(Clerck, 1757)

##### Distribution

Palearctic.

##### Notes

Previously recorded from unspecified locality between Resen and Ohrid (Drensky 1929, Drensky 1936).

#### Alopecosa inquilina

(Clerck, 1757)

##### Distribution

Palearctic.

##### Notes

Previously recorded from unspecified locality between Resen and Ohrid (Drensky 1929, Drensky 1936).

#### Alopecosa mariae

(Dahl, 1908)

##### Materials

- **Type status:** Other material. **Occurrence:** recordedBy: C. Deltshev & M. Komnenov; sex: 1 female; **Location:** country: FYR of Macedonia; locality: Galichitsa Mt., Tomoros peak; verbatimElevation: 1830 m; **Event:** eventDate: 22-06-2008

##### Distribution

Palearctic.

##### Notes

Previously recorded from Galichitsa, Preseka, 1600 m (Lazarov 2004).

#### Alopecosa pulverulenta

(Clerck, 1757)

##### Distribution

Palearctic.

##### Notes

Previously reciorded from Ohrid, Studenchitsa (Drensky 1929, Drensky 1936).

#### Alopecosa simoni

(Thorell, 1872)

##### Distribution

Mediterranean.

##### Notes

Previously recorded from Ohrid, Studenchitsa (Drensky 1929, Drensky 1936).

#### Alopecosa sulzeri

(Pavesi, 1873)

##### Distribution

Palearctic.

##### Notes

Previously recorded from Ohrid (Drensky 1929, Drensky 1936)

#### Alopecosa trabalis

(Clerck, 1757)

##### Distribution

Palearctic.

##### Notes

Previously recorded from unspecified locality between Resen and Ohrid (Drensky 1929, Drensky 1936).

#### Arctosa leopardus

(Sundevall, 1833)

##### Distribution

Palearctic.

##### Notes

Previously recorded from unspecified locality between Resen and Ohrid (Drensky 1929, Drensky 1936).

#### Arctosa maculata

(Hahn, 1822)

##### Distribution

European.

##### Notes

Previously recorded from unspecified locality between Resen and Ohrid (Drensky 1929, Drensky 1936).

#### Arctosa perita

(Latreille, 1799)

##### Distribution

Holarctic.

##### Notes

Previously recorded from Ohrid (Drensky 1929).

#### Aulonia albimana

(Walckenaer, 1805)

##### Distribution

Palearctic.

##### Notes

Previously recorded from Ohrid, Studenchitsa (Drensky 1929, Drensky 1936).

#### Lycosa tarantula

(Linnaeus, 1758)

##### Distribution

North Mediterranean.

##### Notes

Previously recorded from unspecified locality between Ohrid and Resen (Drensky 1929, Drensky 1936).

#### Pardosa agrestis

(Westring, 1861)

##### Distribution

Palearctic.

##### Notes

Previously recorded from Ohrid (Drensky 1929, Drensky 1936).

#### Pardosa agricola

(Thorell, 1856)

##### Distribution

Europeo-Central Asiatic.

##### Notes

Previously recorded from unspecified locality between Resen and Ohrid (Drensky 1929).

#### Pardosa albatula

(Roewer, 1951)

##### Materials

- **Type status:** Other material. **Occurrence:** recordedBy: C. Deltshev & M. Komnenov; sex: 3 females; **Location:** country: FYR of Macedonia; locality: Galichitsa Mt., Dzhafa pool; verbatimElevation: 1650 m; **Event:** eventDate: 17-06-2008

##### Distribution

European.

##### Notes

Previously recorded from unspecified locality between Resen and Ohrid (Drensky 1929, Drensky 1936).

#### Pardosa amentata

(Clerck, 1757)

##### Distribution

European.

##### Notes

Previously recorded from unspecified locality between Resen and Ohrid (Drensky 1929, Drensky 1936).

#### Pardosa atomaria

(C. L. Koch, 1847)

##### Distribution

Northeast Mediterranean.

##### Notes

Previously recorded from unspecified locality between Resen and Ohrid (Drensky 1929, Drensky 1936, Nikolić and Polenec 1981).

#### Pardosa blanda

(C. L. Koch, 1833)

##### Distribution

Palearctic.

##### Notes

Previously recorded from Preseka (Drensky 1929, Drensky 1936, Nikolić and Polenec 1981).

#### Pardosa hortensis

(Thorell, 1872)

##### Materials

- **Type status:** Other material. **Occurrence:** recordedBy: C. Deltshev & M. Komnenov; sex: 1 female; **Location:** country: FYR of Macedonia; locality: Galichitsa Mt., Stenje vill., Stenjsko Blato bog; verbatimElevation: 850 m; **Event:** eventDate: 17-06-2008

##### Distribution

Palearctic.

##### Notes

Previously recorded from Ohrid (Stojićević 1929).

#### Pardosa lugubris

(Walckenaer, 1802)

##### Materials

- **Type status:** Other material. **Occurrence:** recordedBy: D. Vidincheva; **Location:** country: FYR of Macedonia; locality: Galichitsa Mt.; verbatimElevation: 600-1800 m; **Event:** eventDate: 26-10-1992

##### Distribution

Palearctic.

##### Notes

Previously recorded from Ohrid (Drensky 1929, Drensky 1936).

#### Pardosa monticola

(Clerck, 1757)

##### Distribution

Palearctic.

##### Notes

Previously recorded from unspecified locality between Resen and Ohrid (Drensky 1929, Drensky 1936)

#### Pardosa riparia

(C. L. Koch, 1833)

##### Distribution

Palearctic.

##### Notes

Unspecified locality between Resen and Ohrid (Drensky 1929).

#### Pardosa paludicola

(Clerck, 1757)

##### Distribution

Palearctic.

##### Notes

Ohrid, Studenchitsa (Stojićević 1929).

#### Pardosa palustris

(Linnaeus, 1758)

##### Distribution

Holarctic.

##### Notes

Previously recorded from unspecified locality between Ohrid and Resen (Drensky 1929, Drensky 1936).

#### Pardosa prativaga

(L. Koch, 1870)

##### Materials

- **Type status:** Other material. **Occurrence:** recordedBy: C. Deltshev & M. Komnenov; sex: 1 female; **Location:** country: FYR of Macedonia; locality: Galichitsa Mt., Stenje vill., Stenjsko Blato bog; verbatimElevation: 850 m; **Event:** eventDate: 17-06-2008

##### Distribution

European.

##### Notes

First record in Galichitsa Mt.

#### Pardosa pullata

(Clerck, 1757)

##### Materials

- **Type status:** Other material. **Occurrence:** recordedBy: C. Deltshev & M. Komnenov; sex: 1 male, 1 female; **Location:** country: FYR of Macedonia; locality: Galichitsa Mt., Stenje vill., Stenjsko Blato bog; verbatimElevation: 850 m; **Event:** eventDate: 18-06-2008

##### Distribution

Europeo-Central Asiatic.

##### Notes

Previously recorded from unspecified locality between Ohrid and Resen (Drensky 1929, Drensky 1936).

#### Pardosa vittata

(Keyserling, 1863)

##### Distribution

European.

##### Notes

Unspecified locality between Resen and Ohrid (Drensky 1929, Drensky 1936).

#### Pirata knorri

(Scopoli, 1763)

##### Distribution

Palearctic.

##### Notes

Previously recorded from unspecified locality between Resen and Ohrid (Drensky 1929, Drensky 1936).

#### Pirata latitans

(Blackwall, 1841)

##### Materials

- **Type status:** Other material. **Occurrence:** recordedBy: C. Deltshev & M. Komnenov; sex: 1 female; **Location:** country: FYR of Macedonia; locality: Galichitsa Mt., Stenje vill., Stenjsko Blato bog; verbatimElevation: 850 m; **Event:** eventDate: 18-06-2008

##### Distribution

European.

##### Notes

First record in Galichitsa Mt.

#### Pirata piscatorius

(Clerck, 1757)

##### Distribution

Palearctic.

##### Notes

Previously recorded from unspecified locality between Resen and Ohrid (Drensky 1929, Drensky 1936).

#### Trochosa hispanica

Simon, 1870

##### Materials

- **Type status:** Other material. **Occurrence:** recordedBy: C. Deltshev & M. Komnenov; sex: 1 female; **Location:** country: FYR of Macedonia; locality: Galichitsa Mt., Stenje vill., Stenjsko Blato bog; verbatimElevation: 850 m; **Event:** eventDate: 17-06-2008

##### Distribution

Mediterranean.

##### Notes

First record in Galichitsa Mt.

#### Trochosa ruricola

(De Geer, 1778)

##### Distribution

Holarctic.

##### Notes

Previously recorded from Ohrid, Studenchitsa (Drensky 1929, Drensky 1936).

#### Trochosa terricola

Thorell, 1856

##### Distribution

Holarctic.

##### Notes

Previously recorded from unspecified locality between Resen and Ohrid (Drensky 1929, Drensky 1936).

#### Xerolycosa nemoralis

(Westring, 1861)

##### Materials

- **Type status:** Other material. **Occurrence:** recordedBy: C. Deltshev & M. Komnenov; sex: 1 male; **Location:** country: FYR of Macedonia; locality: Galichitsa Mt., Stenje vill., Stenjsko Blato bog; verbatimElevation: 850 m; **Event:** eventDate: 17-06-2008

##### Distribution

Palearctic.

##### Notes

Previously recorded from unspecified locality between Ohrid and Resen (Drensky 1929, Drensky 1936).

#### PISAURIDAE

#### Dolomedes fimbriatus

(Clerck, 1757)

##### Distribution

Palearctic.

##### Notes

Previously recorded from Ohrid (Drensky 1929, Drensky 1936).

#### Pisaura mirabilis

(Clerck, 1757)

##### Materials

- **Type status:** Other material. **Occurrence:** recordedBy: C. Deltshev & M. Komnenov; sex: 1 juv.; **Location:** country: FYR of Macedonia; locality: Galichitsa Mt., Crvena Lokva pool; verbatimElevation: 1620 m; **Event:** eventDate: 20-06-2008
- **Type status:** Other material. **Occurrence:** recordedBy: C. Deltshev & M. Komnenov; sex: 1 female; **Location:** country: FYR of Macedonia; locality: Galichitsa Mt., Dzhafa pool; verbatimElevation: 1650 m; **Event:** eventDate: 18-06-2008
- **Type status:** Other material. **Occurrence:** recordedBy: C. Deltshev & M. Komnenov; sex: 1 female; **Location:** country: FYR of Macedonia; locality: Galichitsa Mt., Stenje vill., Stenjsko Blato bog; verbatimElevation: 850 m; **Event:** eventDate: 17-06-2008

##### Distribution

Palearctic.

##### Notes

First record in Galichitsa Mt.

#### OXYOPIDAE

#### Oxyopes heterophthalmus

(Latreille, 1804)

##### Materials

- **Type status:** Other material. **Occurrence:** recordedBy: C. Deltshev & M. Komnenov; sex: 2 females; **Location:** country: FYR of Macedonia; locality: Galichitsa Mt., Dzhafa pool; verbatimElevation: 1650 m; **Event:** eventDate: 18-06-2008
- **Type status:** Other material. **Occurrence:** recordedBy: C. Deltshev & M. Komnenov; sex: 1 female; **Location:** country: FYR of Macedonia; locality: Galichitsa Mt., Stenje vill., Stenjsko Blato bog; verbatimElevation: 850 m; **Event:** eventDate: 17-06-2008
- **Type status:** Other material. **Occurrence:** recordedBy: C. Deltshev & M. Komnenov; sex: 1 female; **Location:** country: FYR of Macedonia; locality: Galichitsa Mt., Tomoros peak; verbatimElevation: 1830 m; **Event:** eventDate: 22-06-2008
- **Type status:** Other material. **Occurrence:** recordedBy: C. Deltshev & M. Komnenov; sex: 1 female; **Location:** country: FYR of Macedonia; locality: Galichitsa Mt., vill. Leskovec, Leskovska Peshtera cave; verbatimElevation: 1066 m; **Event:** eventDate: 18-06-2008

##### Distribution

Palearctic.

##### Notes

Previously recorded from unspecified locality between Resen and Ohrid (Drensky 1929, Drensky 1936).

#### Oxyopes lineatus

Latreille, 1806

##### Materials

- **Type status:** Other material. **Occurrence:** recordedBy: C. Deltshev & M. Komnenov; sex: 1 male; **Location:** country: FYR of Macedonia; locality: Galichitsa Mt., Dzhafa pool; verbatimElevation: 1650 m; **Event:** eventDate: 18-06-2008
- **Type status:** Other material. **Occurrence:** recordedBy: C. Deltshev & M. Komnenov; sex: 1 female; **Location:** country: FYR of Macedonia; locality: Galichitsa Mt., Tomoros peak; verbatimElevation: 1830 m; **Event:** eventDate: 22-06-2008

##### Distribution

Palearctic.

##### Notes

Previously recorded from unspecified locality between Resen and Ohrid (Drensky 1929, Drensky 1936).

#### ZORIDAE

#### Zora prespaensis

Drenski, 1929

##### Materials

- **Type status:** Other material. **Occurrence:** recordedBy: C. Deltshev & M. Komnenov; sex: 1 female; **Location:** country: FYR of Macedonia; locality: Galichitsa Mt., Stenje vill., Stenjsko Blato bog; verbatimElevation: 850 m; **Event:** eventDate: 18-06-2008

##### Distribution

Galichitsa endemic.

##### Notes

Previously recorded from Prespa Lake, Resen (Drensky 1929, Drensky 1936)

#### AGELENIDAE

#### Agelena labyrinthica

(Clerck, 1757)

##### Distribution

Palearctic.

##### Notes

Previously recorded from Preseka: (Lazarov 2004).

#### Allagelena gracilens

C.L. Koch, 1841

##### Materials

- **Type status:** Other material. **Occurrence:** recordedBy: C. Deltshev & G. Blagoev; **Location:** country: FYR of Macedonia; locality: Galichitsa Mt., Ohrid, Studenchitsa; verbatimElevation: 695 m; **Event:** eventDate: 30-08-2002

##### Distribution

Mediterrano-Central Asiatic.

##### Notes

First record in Galichitsa Mt.

#### Inermocoelotes falciger

Kulczyński, 1897

##### Materials

- **Type status:** Other material. **Occurrence:** recordedBy: D. Vidincheva; **Location:** country: FYR of Macedonia; locality: Galichitsa Mt.; verbatimElevation: 600-1800 m; **Event:** eventDate: 26-10-1992

##### Distribution

Southeast European.

##### Notes

First record in Galichitsa Mt.

#### Inermocoelotes karlinskii

(Kulczynski, 1906)

##### Distribution

Southeast European.

##### Notes

Previously recorded from Ohrid (Drensky 1929).

#### Malthonica campestris

C. L. Koch, 1834

##### Distribution

European.

##### Notes

Previously recorded from unspecified locality between Resen and Ohrid (Drensky 1929, Drensky 1936).

#### Malthonica ferruginea

(Panzer, 1804)

##### Materials

- **Type status:** Other material. **Occurrence:** recordedBy: C. Deltshev & E. Stojkoska; sex: 1 female; **Location:** country: FYR of Macedonia; locality: Galichitsa Mt., Ohrid, Sv. Stefan; verbatimElevation: 680 m; **Event:** eventDate: 31-08-2005

##### Distribution

European.

##### Notes

Previously recorded from unspecified locality between Ohrid and Resen (Drensky 1929, Drensky 1936).

#### Malthonica nemorosa

Simon, 1916

##### Distribution

North Mediterranean.

##### Notes

Previuosly recorded from Ohrid, Lagadin (Deltshev et al. 2000).

#### Malthonica silvestris

C. L. Koch, 1872

##### Distribution

European.

##### Notes

Previously recorded from Resen (Drensky 1929, Drensky 1936).

#### Tegenaria atrica

C. L. Koch, 1843

##### Distribution

European.

##### Notes

Previously recorded from Ohrid (Stojićević 1929).

#### Tegenaria domestica

(Clerck, 1757)

##### Materials

- **Type status:** Other material. **Occurrence:** recordedBy: C. Deltshev, M. Komnenov; sex: 1 female; **Location:** country: FYR of Macedonia; locality: Galichitsa Mt., Golem Grad island; verbatimElevation: 842 m; **Event:** eventDate: 20-06-2008
- **Type status:** Other material. **Occurrence:** recordedBy: C. Deltshev, M. Komnenov, E. Stojkoska; sex: 2 juv; **Location:** country: FYR of Macedonia; locality: Galichitsa Mt., Ohrid, Trpejca vill, cave; verbatimElevation: 940 m; **Event:** eventDate: 09-12-10

##### Distribution

Cosmopolitan.

#### Tegenaria paragamiani

Deltshev, 2008

##### Materials

- **Type status:** Other material. **Occurrence:** recordedBy: C. Deltshev & M. Komnenov; sex: 1 male, 1 female; **Location:** country: FYR of Macedonia; locality: Galichitsa Mt., Ohrid, Mechkina Dupka cave; verbatimElevation: 1020 m; **Event:** eventDate: 20-06-2008

##### Distribution

Balkan endemic.

##### Notes

First record in FYR of Macedonia.

#### Tegenaria parietina

(Fourcroy, 1785)

##### Distribution

West Palearctic.

##### Notes

Previously recorded from unspecified locality between Ohrid and Resen (Drensky 1929, Drensky 1936).

#### Tegenaria regispyrrhi

Brignoli, 1976

##### Materials

- **Type status:** Other material. **Occurrence:** recordedBy: C. Deltshev & M. Komnenov; sex: 1 female; **Location:** country: FYR of Macedonia; locality: Galichitsa Mt., Tomoros Peak; verbatimElevation: 1830 m; **Event:** eventDate: 22-06-2008

##### Distribution

Balkan endemic.

##### Notes

First record in FYR of Macedonia.

#### CYBAEIDAE

#### Argyroneta aquatica

(Clerck, 1757)

##### Distribution

Palearctic.

##### Notes

Previously recorded from Resen (Drensky 1929, Drensky 1936).

#### Cybaeus angustiarum

L. Koch, 1868

##### Distribution

European.

##### Notes

Previously recorded from unspecified locality between Resen and Ohrid (Drensky 1929, Drensky 1936).

#### DICTYNIDAE

#### Dictyna arundinacea

(Linnaeus, 1758)

##### Materials

- **Type status:** Other material. **Occurrence:** recordedBy: C. Deltshev & E. Stojkoska; sex: 2 females; **Location:** country: FYR of Macedonia; locality: Galichitsa Mt., Stenje vill., Stenjsko Blato bog; verbatimElevation: 850 m; **Event:** eventDate: 30-08-2005

##### Distribution

Holarctic.

##### Notes

Previously recorded from Ohrid (Drensky 1929, Drensky 1936, Stojićević 1929).

#### Dictyna civica

(Lucas, 1850)

##### Materials

- **Type status:** Other material. **Location:** country: FYR of Macedonia; locality: Galichitsa Mt., Ohrid, Sv. Stefan; verbatimElevation: 680 m; **Event:** eventDate: 17-06-2008

##### Distribution

Holarctic.

##### Notes

First record in Galichitsa Mt.

#### Dictyna latens

(Fabricius, 1775)

##### Materials

- **Type status:** Other material. **Occurrence:** recordedBy: C. Deltshev & M. Komnenov; sex: 1 male, 3 females; **Location:** country: FYR of Macedonia; locality: Galichitsa Mt., Dzhafa pool; verbatimElevation: 1650 m; **Event:** eventDate: 18-06-2008

##### Distribution

Europeo-Central Asiatic.

##### Notes

First record in FYR of Macedonia.

#### Dictyna pusilla

Thorell, 1856

##### Materials

- **Type status:** Other material. **Occurrence:** recordedBy: C. Deltshev & M. Komnenov; sex: 1 female; **Location:** country: FYR of Macedonia; locality: Galichitsa Mt., Stenje vill., Stenjsko Blato bog; verbatimElevation: 850 m; **Event:** eventDate: 18-06-2008

##### Distribution

Palearctic.

##### Notes

First record in Galichitsa Mt.

#### Dictyna uncinata

Thorell, 1856

##### Distribution

Palearctic.

##### Notes

Previously recorded from Ohrid and Resen (Drensky 1929, Drensky 1936).

#### Dictyna vicina

Simon, 1873

##### Distribution

Mediterranean.

##### Notes

Previously recorded from Ohrid (Drensky 1929, Drensky 1936).

#### AMAUROBIIDAE

#### Amaurobius fenestralis

(Ström, 1768)

##### Distribution

European.

##### Notes

Previously recorded from unspecified locality between Resen and Ohrid (Drensky 1929, Drensky 1936).

#### Amaurobius pallidus

L. Koch, 1868

##### Distribution

Southeast European.

##### Notes

Previously recorded from Ohrid (Drensky 1929, Drensky 1936).

#### TITANOECIDAE

#### Nurscia albomaculata

(Lucas, 1846)

##### Distribution

Europeo-Central Asiatic.

##### Notes

Previously recorded from unspecified locality between Resen and Ohrid (Drensky 1929, Drensky 1936).

#### MITURGIDAE

#### Cheiracanthium elegans

Thorell, 1875

##### Materials

- **Type status:** Other material. **Occurrence:** recordedBy: C. Deltshev & M. Komnenov; sex: 1 female; **Location:** country: FYR of Macedonia; locality: Galichitsa Mt., Tomoros peak; verbatimElevation: 1830 m; **Event:** eventDate: 22-06-2008
- **Type status:** Other material. **Occurrence:** recordedBy: C. Deltshev & M. Komnenov; sex: 1 male, 1 female; **Location:** country: FYR of Macedonia; locality: Galichitsa Mt., Zhichara; verbatimElevation: 1515 m; **Event:** eventDate: 20-06-2008

##### Distribution

European.

##### Notes

Previously recorded from unspecified locality between Resen and Ohrid (Drensky 1929, Drensky 1936)

#### Cheiracanthium ienisteai

Stergiu, 1985

##### Materials

- **Type status:** Other material. **Occurrence:** recordedBy: C. Deltshev & M. Komnenov; sex: 1 female; **Location:** country: FYR of Macedonia; locality: Galichitsa Mt., vill. Stenje, Stenjsko Blato bog; verbatimElevation: 850 m; **Event:** eventDate: 17-06-2008

##### Distribution

Southeast European.

##### Notes

First record in FYR of Macedonia.

#### Cheiracanthium erraticum

(Walckenaer, 1802)

##### Distribution

Palearctic.

##### Notes

Previously recorded from Ohrid (Drensky 1929, Drensky 1936).

#### Cheiracanthium mildei

L. Koch, 1864

##### Distribution

Holarctic.

##### Notes

Previously recorded from Ohrid (Drensky 1929, Drensky 1936).

#### Cheiracanthium macedonicum

Drenski, 1921

##### Distribution

Balkan endemic.

##### Notes

Previously recorded from unspecified locality between Resen and Ohrid (Drensky 1929, Drensky 1936).

#### Cheiracanthium seidlitzi

L. Koch, 1864

##### Distribution

Mediterranean.

##### Notes

Previously recorded from Ohrid (Drensky 1929, Drensky 1936).

#### LIOCRANIDAE

#### Agroeca cuprea

Menge, 1873

##### Materials

- **Type status:** Other material. **Occurrence:** recordedBy: D. Vidincheva; **Location:** country: FYR of Macedonia; locality: Galichitsa Mt.; verbatimElevation: 600-1800 m; **Event:** eventDate: 26-10-1992

##### Distribution

Palearctic.

##### Notes

First record in Galichitsa Mt.

#### Liocranoecea striata

Kulczyński, 1882

##### Distribution

Europeo-Central Asiatic.

##### Notes

Previously recorded from unspecified locality between Resen and Ohrid (Drensky 1929, Drensky 1936).

#### Liocranum rupicola

(Walckenaer, 1830)

##### Materials

- **Type status:** Other material. **Occurrence:** sex: 1 female; **Location:** country: FYR of Macedonia; locality: Galichitsa Mt., Peshtani vill.; verbatimElevation: 719 m; **Event:** eventDate: 30-08-2005
- **Type status:** Other material. **Occurrence:** sex: 1 female; **Location:** country: FYR of Macedonia; locality: Galichitsa Mt., Stenje vill.; verbatimElevation: 850 m; **Event:** eventDate: 31-08-2005

##### Distribution

European.

##### Notes

First record in Galichitsa Mt.

#### Sagana rutilans

(Thorell, 1875)

##### Distribution

European.

##### Notes

Previously recorded from Ohrid, Studenchitsa (Drensky 1929, Drensky 1936).

#### CLUBIONIDAE

#### Clubiona brevipes

Blackwall, 1841

##### Materials

- **Type status:** Other material. **Occurrence:** recordedBy: D. Vidincheva; **Location:** country: FYR of Macedonia; locality: Galichitsa Mt.; verbatimElevation: 600-1800 m; **Event:** eventDate: 26-10-1992

##### Distribution

Palearctic.

##### Notes

First record in Galichitsa Mt.

#### Clubiona comta

C. L. Koch, 1839

##### Distribution

West Palearctic.

##### Notes

Previously recorded from Ohrid (Drensky 1929, Drensky 1936).

#### Clubiona genevensis

L. Koch, 1866

##### Materials

- **Type status:** Other material. **Occurrence:** recordedBy: B. Petrov; sex: 1 male; **Location:** country: FYR of Macedonia; locality: Galichitsa Mt., Otechevo; verbatimElevation: 1609-1650 m; **Event:** eventDate: 01-05-2008

##### Distribution

Palearctic.

##### Notes

First record for FYR Macedonia.

#### Clubiona hilaris

Simon, 1878

##### Distribution

European.

##### Notes

Previously recorded from Resen and Ohrid (Drensky 1929, Drensky 1936).

#### Clubiona marmorata

L. Koch, 1866

##### Distribution

European.

##### Notes

Previously recorded from Ohrid (Drensky 1929).

#### Clubiona neglecta

O. P. Cambridge, 1862

##### Distribution

Palearctic.

##### Notes

Previously recorded from Ohrid (Drensky 1929, Drensky 1936).

#### Clubiona pallidula

(Clerck, 1757)

##### Distribution

Holarctic.

##### Notes

Previously recorded from Ohrid (Drensky 1929, Drensky 1936).

#### CORINNIDAE

#### Cetonana laticeps

(Canestrini, 1868)

##### Distribution

European.

##### Notes

Previously recorded from Ohrid, Lagadin (Deltshev et al. 2000).

#### Araneae

#### Phrurolithus corsicus

(Simon, 1878)

##### Distribution

European.

##### Notes

Previously recorded from unspecified locality between Resen and Ohrid (Drensky 1929, Drensky 1936).

#### Phrurulitus festivus

(C. L. Koch, 1835)

##### Materials

- **Type status:** Other material. **Occurrence:** recordedBy: C. Deltshev & M. Komnenov; sex: 1 male; **Location:** country: FYR of Macedonia; locality: Galichitsa Mt., Dzhafa pool; verbatimElevation: 1650 m; **Event:** eventDate: 18-06-2008
- **Type status:** Other material. **Occurrence:** recordedBy: P. Beron; **Location:** country: FYR of Macedonia; locality: Galichitsa Mt., Ohrid; verbatimElevation: 1050 m; **Event:** eventDate: 18-05-1993

##### Distribution

Palearctic.

##### Notes

Previously recotded from Preseka (Lazarov 2004).

#### Phrurolithus pullatus

Kulczyński, 1897

##### Distribution

Europeo-Central Asiatic.

##### Notes

Previously recorded from unspecified locality between Resen and Ohrid (Drensky 1929, Drensky 1936).

#### Phrurolithus szilyi

Herman, 1879

##### Materials

- **Type status:** Other material. **Occurrence:** recordedBy: C. Deltshev & M. Komnenov; sex: 1 female; **Location:** country: FYR of Macedonia; locality: Galichitsa Mt., Dzhafa pool; verbatimElevation: 1650 m; **Event:** eventDate: 17-06-2008

##### Distribution

European.

##### Notes

First record in Galichitsa Mt.

#### ZODARIIDAE

#### Zodarion gallicum

(Simon, 1873)

##### Distribution

North Mediterranean.

##### Notes

Previously recorded from Ohrid (Stojićević 1929).

#### Zodarion italicum

(Canestrini, 1868)

##### Distribution

European.

##### Notes

Previously recorded from Ohrid (Drensky 1929, Drensky 1936).

#### Zodarion ohridense

Wunderlich, 1973

##### Materials

- **Type status:** Other material. **Occurrence:** recordedBy: C. Deltshev & G. Blagoev; sex: 1 male; **Location:** country: FYR of Macedonia; locality: Galichitsa Mt., Ohrid, Studenchitsa; verbatimElevation: 690 m; **Event:** eventDate: 30-08-2002
- **Type status:** Other material. **Occurrence:** recordedBy: C. Deltshev & M. Komnenov; sex: 1 female; **Location:** country: FYR of Macedonia; locality: Galichitsa Mt., Zhichara; verbatimElevation: 1515 m; **Event:** eventDate: 20-06-2008

##### Distribution

Balkan endemic.

##### Notes

Previously ecorded from Ohrid (Wunderlich 1973).

#### GNAPHOSIDAE

#### Callilepis nocturna

(Linnaeus, 1758)

##### Materials

- **Type status:** Other material. **Occurrence:** recordedBy: C. Deltshev & M. Komnenov; sex: 1 female; **Location:** country: FYR of Macedonia; locality: Galichitsa Mt., Dzhafa pool; verbatimElevation: 1650 m; **Event:** eventDate: 17-06-2008
- **Type status:** Other material. **Occurrence:** recordedBy: C. Deltshev & M. Komnenov; sex: 1 female; **Location:** country: FYR of Macedonia; locality: Galichitsa Mt., Zhichara; verbatimElevation: 1515 m; **Event:** eventDate: 20-06-2008

##### Distribution

Palearctic.

##### Notes

Previously recorded from Ohrid, Studenchtsa (Drensky 1929, Drensky 1936).

#### Callilepis schuszteri

(Herman, 1879)

##### Materials

- **Type status:** Other material. **Occurrence:** recordedBy: C. Deltshev & M. Komnenov; sex: 1 female; **Location:** country: FYR of Macedonia; locality: Galichitsa Mt., Simoncheska Lokva pool; verbatimElevation: 1680 m; **Event:** eventDate: 18-06-2008

##### Distribution

Palearctic.

##### Notes

First record in FYR of Macedonia.

#### Drassodes lapidosus

(Walckenaer, 1802)

##### Materials

- **Type status:** Other material. **Occurrence:** recordedBy: C. Deltshev & G. Blagoev; sex: 1 female; **Location:** country: FYR of Macedonia; locality: Galichitsa Mt., Bugarska Chuka Peak; verbatimElevation: 1797 m; **Event:** eventDate: 19-06-2008
- **Type status:** Other material. **Occurrence:** recordedBy: C. Deltshev & G. Blagoev; sex: 1 male; **Location:** country: FYR of Macedonia; locality: Galichitsa Mt., Dzhafa pool; verbatimElevation: 1650 m; **Event:** eventDate: 17-06-2008
- **Type status:** Other material. **Occurrence:** recordedBy: C. Deltshev & G. Blagoev; sex: 1 male; **Location:** country: FYR of Macedonia; locality: Galichitsa Mt., Resen; verbatimElevation: 1000 m; **Event:** eventDate: 30-08-2002
- **Type status:** Other material. **Occurrence:** recordedBy: C. Deltshev & M. Komnenov; sex: 1 male; **Location:** country: FYR of Macedonia; locality: Galichitsa Mt., Stenje vill., Stenjsko Blato bog; verbatimElevation: 850 m; **Event:** eventDate: 17-06-2008

##### Distribution

Palearctic.

##### Notes

Previously recorded from Resen and Ohrid, Srudenchitsa (Drensky 1929, Drensky 1936, Stojićević 1929).

#### Drassodes pubescens

(Thorell, 1856)

##### Distribution

Palearctic.

##### Notes

Previously recorded from Preseka (Lazarov 2004).

#### Drassyllus praeficus

(L. Koch, 1866)

##### Materials

- **Type status:** Other material. **Occurrence:** recordedBy: C. Deltshev & M. Komnenov; sex: 2 females; **Location:** country: FYR of Macedonia; locality: Galichitsa Mt., Bugarska Chuka Peak; verbatimElevation: 1797 m; **Event:** eventDate: 19-06-2008

##### Distribution

Europeo-Central Asiatic.

##### Notes

Previously recorded from unspecified locality between Resen and Ohrid (Drensky 1929, Drensky 1936).

#### Drassyllus villicus

(Thorell, 1875)

##### Materials

- **Type status:** Other material. **Occurrence:** recordedBy: D. Vidincheva; **Location:** country: FYR of Macedonia; locality: Galichitsa Mt.; verbatimElevation: 600-1800 m; **Event:** eventDate: 26-10-1992

##### Distribution

Palearctic.

##### Notes

First record in Galichitsa Mt.

#### Gnaphosa bicolor

(Hahn, 1833)

##### Materials

- **Type status:** Other material. **Occurrence:** recordedBy: C. Deltshev & M. Komnenov; sex: 1 female; **Location:** country: FYR of Macedonia; locality: Galichitsa Mt., Simoncheska Lokva pool; verbatimElevation: 1680 m; **Event:** eventDate: 18-06-2008

##### Distribution

European.

##### Notes

Previously recorded from Galichitsa Mt (Drensky 1929, Drensky 1936).

#### Gnaphosa dolosa

Herman, 1879

##### Distribution

Palearctic.

##### Notes

Previously recorded from Ohrid (Drensky 1929, Drensky 1936).

#### Gnaphosa tetrica

Simon, 1878

##### Distribution

North Mediterranean.

##### Notes

Previously recorded from unspecified locality between Resen and Ohrid (Drensky 1936, Drensky 1929).

#### Gnaphosa lucifuga

(Wilckenaer, 1802)

##### Distribution

Palearctic.

##### Notes

Previously recorded from Preseka (Drensky 1929, Drensky 1936, Lazarov 2004).

#### Gnaphosa montana

(L. Koch, 1866)

##### Distribution

Palearctic.

##### Notes

Previously recorded from Ohrid and Resen (Drensky 1929, Drensky 1936).

#### Haplodrassus signifer

(C. L. Koch, 1839)

##### Distribution

Holarctic.

##### Notes

Previously recorded from Galichitsa Mt (Drensky 1929, Drensky 1936).

#### Haplodrassus silvestris

(Blackwall, 1833)

##### Materials

- **Type status:** Other material. **Occurrence:** recordedBy: D. Vidincheva; **Location:** country: FYR of Macedonia; locality: Galichitsa Mt.; verbatimElevation: 600-1800 m; **Event:** eventDate: 26-10-1992

##### Distribution

Palearctic.

##### Notes

First record in Galichitsa Mt.

#### Micaria albovittata

(Lucas, 1846)

##### Materials

- **Type status:** Other material. **Occurrence:** recordedBy: C. Deltshev & M. Komnenov; sex: 1 male; **Location:** country: FYR of Macedonia; locality: Galichitsa Mt., Simoncheska Lokva pool; verbatimElevation: 1680 m; **Event:** eventDate: 18-06-2008

##### Distribution

Palearctic.

##### Notes

First record in Galichitsa Mt.

#### Nomisia aussereri

(C. L. Koch, 1872)

##### Materials

- **Type status:** Other material. **Occurrence:** recordedBy: C. Deltshev & E. Stojkoska; sex: 4 females; **Location:** country: FYR of Macedonia; locality: Galichitsa Mt., Stenje vill., Stenjsko Blato bog; verbatimElevation: 850 m; **Event:** eventDate: 30-08-2005

##### Distribution

Palearctic.

##### Notes

First record in Galichitsa Mt.

#### Nomisia exornata

(C.L. Koch, 1839)

##### Materials

- **Type status:** Other material. **Occurrence:** recordedBy: C. Deltshev & G. Blagoev; sex: 1 male; **Location:** country: FYR of Macedonia; locality: Galichitsa Mt., Resen; verbatimElevation: 1000 m; **Event:** eventDate: 30-08-2002

##### Distribution

Europeo-Central Asiatic.

##### Notes

First record in Galichitsa Mt.

#### Scotophaeus scutulatus

(L. Koch, 1866)

##### Distribution

West Palearctic.

##### Notes

Previously recorded from Petrinska planina, Resen (Drensky 1929, Drensky 1936).

#### Setaphis carmeli

(O. P.-Cambridge, 1872)

##### Distribution

Mediterranean.

##### Notes

Previously recorded from Ohrid, Studenchitsa (Drensky 1929).

#### Trachyzelotes pedestris

(C. L. Koch, 1837)

##### Distribution

European.

##### Notes

Unspecified locality between Resen and Ohrid (Drensky 1929, Drensky 1936).

#### Zelotes apricorum

(L. Koch, 1876)

##### Materials

- **Type status:** Other material. **Occurrence:** recordedBy: C. Deltshev & G. Blagoev; sex: 1 male; **Location:** country: FYR of Macedonia; locality: Galichitsa Mt., Resen; verbatimElevation: 1000 m; **Event:** eventDate: 30-08-2002

##### Distribution

European.

##### Notes

First record in Galichitsa Mt.

#### Zelotes babunaensis

(Drensky, 1829)

##### Distribution

Balkan endemic.

##### Notes

Previously recorded from Ohrid, Resen (Drensky 1929, Drensky 1936).

#### Zelotes caucasius

(L. Koch, 1866)

##### Distribution

Europeo-Central Asiatic.

##### Notes

Previously recorded from Ohrid (Drensky 1929, Drensky 1936, Stojićević 1929).

#### Zelotes clivicola

(L. Koch, 1870)

##### Distribution

Palearctic.

##### Notes

Previously recorded from Ohrid (Stojićević 1929).

#### Zelotes gracilis

(Canestini, 1868)

##### Distribution

Middle and Southeast European.

##### Notes

Previously recorded from unspecified locality between Resen and Ohrid (Drensky 1929, Drensky 1936).

#### Zelotes manius

(Simon, 1878)

##### Distribution

East European.

##### Notes

Previously recorded from unspecified locality between Resen and Ohrid (Drensky 1929, Drensky 1936).

#### Zelotes oblongus

(C.L. Koch, 1833)

##### Materials

- **Type status:** Other material. **Occurrence:** recordedBy: C. Deltshev & E. Stojkoska; sex: 1 female; **Location:** country: FYR of Macedonia; locality: Galichitsa Mt., Peshtani vill.; verbatimElevation: 719 m; **Event:** eventDate: 31-08-2005
- **Type status:** Other material. **Occurrence:** recordedBy: C. Deltshev & G. Blagoev; sex: 1 male; **Location:** country: FYR of Macedonia; locality: Galichitsa Mt., Resen; verbatimElevation: 1000 m; **Event:** eventDate: 30-08-2002

##### Distribution

South European.

##### Notes

First record in Galichitsa Mt.

#### Zelotes petrensis

(C. L. Koch, 1839)

##### Materials

- **Type status:** Other material. **Occurrence:** recordedBy: C. Deltshev & E. Stojkoska; sex: 1 female; **Location:** country: FYR of Macedonia; locality: Galichitsa Mt., vill. Stenje, Stenjsko Blato bog; verbatimElevation: 850 m; **Event:** eventDate: 30-08-2005

##### Distribution

Europeo-Central Asiatic.

##### Notes

First record in Galichitsa Mt.

#### Zelotes similis

(Kulczyński, 1887)

##### Materials

- **Type status:** Other material. **Occurrence:** recordedBy: C. Deltshev & M. Komnenov; sex: 3 females; **Location:** country: FYR of Macedonia; locality: Galichitsa Mt., Bugarska Chuka Peak; verbatimElevation: 1797 m; **Event:** eventDate: 19-06-2008
- **Type status:** Other material. **Occurrence:** recordedBy: C. Deltshev & M. Komnenov; **Location:** country: FYR of Macedonia; locality: Galichitsa Mt., Zhichara; verbatimElevation: 1515 m; **Event:** eventDate: 20-06-2008

##### Distribution

European.

##### Notes

First record in FYR of Macedonia.

#### Zelotes subterraneus

(C.L. Koch, 1833)

##### Materials

- **Type status:** Other material. **Occurrence:** recordedBy: D. Vidincheva; **Location:** country: FYR of Macedonia; locality: Galichitsa Mt.; verbatimElevation: 600-1800 m; **Event:** eventDate: 26-10-1992

##### Distribution

Palearctic.

##### Notes

First record in Galichitsa Mt.

#### Zelotes talpinus

(C.L. Koch, 1872)

##### Materials

- **Type status:** Other material. **Occurrence:** recordedBy: C. Deltshev & M. Komnenov; sex: 1 female; **Location:** country: FYR of Macedonia; locality: Galichitsa Mt., Bugarska Chuka peak; verbatimElevation: 1797 m; **Event:** eventDate: 19-06-2008

##### Distribution

Palearctic.

##### Notes

First record in Galichitsa Mt.

#### Zelotes vespertinus

(Thorell, 1875)

##### Distribution

North Mediterranean.

##### Notes

Previously recorded from unspecified locality between Resen and Ohrid (Drensky 1929, Drensky 1936).

#### SPARASSIDAE

#### Micromata virescens

(Clerck, 1757)

##### Materials

- **Type status:** Other material. **Occurrence:** recordedBy: D. Vidincheva; **Location:** country: FYR of Macedonia; locality: Galichitsa Mt.; verbatimElevation: 600-1800 m; **Event:** eventDate: 26-10-1992
- **Type status:** Other material. **Occurrence:** recordedBy: C. Deltshev & E. Stojkoska; sex: 1 female; **Location:** country: FYR of Macedonia; locality: Galichitsa Mt., Zhichara; verbatimElevation: 1500 m; **Event:** eventDate: 30-08-2005

##### Distribution

Palearctic.

##### Notes

First record in Galichitsa Mt.

#### PHILODROMIDAE

#### Philodromus aureolus

(Clerck, 1757)

##### Distribution

Palearctic.

##### Notes

Previously recorded from Resen and Ohrid (Drensky 1929, Drensky 1936).

#### Philodromus cespitum

(Walckenaer, 1802)

##### Materials

- **Type status:** Other material. **Occurrence:** recordedBy: C. Deltshev & M. Komnenov; sex: 1 male, 2 females; **Location:** country: FYR of Macedonia; locality: Galichitsa Mt., Leskovec vill.; verbatimElevation: 1066 m; **Event:** eventDate: 18-06-2008
- **Type status:** Other material. **Occurrence:** recordedBy: C. Deltshev & M. Komnenov; sex: 1 male, 5 females; **Location:** country: FYR of Macedonia; locality: Galichitsa Mt., Stenje vill., Stenjsko Blato bog; verbatimElevation: 850 m; **Event:** eventDate: 17-06-2008

##### Distribution

Holarctic.

##### Notes

First record in Galichitsa Mt.

#### Philodromus laricium

Simon, 1875

##### Distribution

North Mediterranean.

##### Notes

Previously recorded from unspecified locality between Resen and Ohrid (Drensky 1929, Drensky 1936).

#### Philodromus margaritatus

(Clerck, 1757)

##### Distribution

Palearctic.

##### Notes

Previously recorded from unspecified locality between Resen and Ohrid (Drensky 1929).

#### Philodromus poecilus

(Thorell, 1872)

##### Distribution

Palearctic.

##### Notes

Previously recorded from Ohrid (Drensky 1929, Drensky 1936).

#### Philodromus praedatus

O.P.-Cambridge, 1871

##### Materials

- **Type status:** Other material. **Occurrence:** recordedBy: D. Vidincheva; **Location:** country: FYR of Macedonia; locality: Galichitsa Mt.; verbatimElevation: 600-1800 m; **Event:** eventDate: 26-10-1992

##### Distribution

Holarctic.

##### Notes

First record in Galichitsa Mt.

#### Thanatus arenarius

L. Koch, 1872

##### Materials

- **Type status:** Other material. **Occurrence:** recordedBy: C. Deltshev & M. Komnenov; sex: 1 male, 2 females; **Location:** country: FYR of Macedonia; locality: Galichitsa Mt., Stenje vill., Stenjsko Blato bog; verbatimElevation: 850 m; **Event:** eventDate: 17-06-2008

##### Distribution

Europeo-Central Asiatic.

##### Notes

First record in Galichitsa Mt.

#### Thanatus atratus

Simon, 1875

##### Materials

- **Type status:** Other material. **Occurrence:** recordedBy: C. Deltshev & M. Komnenov; sex: 1 male, 2 females; **Location:** country: FYR of Macedonia; locality: Galichitsa Mt., Stenje vill., Stenjsko Blato bog; verbatimElevation: 850 m; **Event:** eventDate: 17-06-2008

##### Distribution

Palearctic.

##### Notes

First record in Galichitsa Mt.

#### Thanatus formicinus

(Clerck, 1757)

##### Distribution

Holarctic.

##### Notes

Previously recorded from Ohrid, Studenchitsa (Drensky 1929).

#### Thanatus lineatipes

Simon, 1870

##### Distribution

Mediterranean.

##### Notes

Previously recorded from unspecified locality between Ohrid and Resen (Drensky 1929, Drensky 1936).

#### Thanatus vulgaris

Simon, 1870

##### Materials

- **Type status:** Other material. **Occurrence:** recordedBy: C. Deltshev & G. Blagoev; sex: 1 male; **Location:** country: FYR of Macedonia; locality: Galichitsa Mt., Resen; verbatimElevation: 1000 m; **Event:** eventDate: 30-08-2002

##### Distribution

Holarctic.

##### Notes

Previously recorded for Galichitsa Mt from Resen (Drensky 1929, Drensky 1936)

#### Tibellus oblongus

(Walckenaer, 1802)

##### Materials

- **Type status:** Other material. **Occurrence:** recordedBy: C. Deltshev & M. Komnenov; sex: 1 female; **Location:** country: FYR of Macedonia; locality: Galichitsa Mt., Stenje vill., Stenjsko Blato bog; verbatimElevation: 850 m; **Event:** eventDate: 18-06-2008

##### Distribution

North Mediterranean.

##### Notes

Previously recorded from Ohrid (Drensky 1929).

#### Tibellus macellus

Simon, 1875

##### Distribution

European.

##### Notes

Previously recorded from unspecified locality between Ohrid and Resen (Drensky 1929, Drensky 1936).

#### THOMISIDAE

#### Diaea dorsata

(Fabricius, 1777)

##### Distribution

Palearctic.

##### Notes

Previously recorded from Galichitsa Mt. (Drensky 1929, Drensky 1936).

#### Ebrechtella tricuspidata

(Fabricius, 1775)

##### Distribution

Palearctic.

##### Notes

Unspecified locality between Resen and Ohrid (Drensky 1929, Drensky 1936).

#### Heriaeus melloteei

Simon, 1884

##### Materials

- **Type status:** Other material. **Occurrence:** recordedBy: C. Deltshev & M. Komnenov; sex: 1 female; **Location:** country: FYR of Macedonia; locality: Galichitsa Mt., Bugarska Chuka Peak; verbatimElevation: 1797 m; **Event:** eventDate: 19-06-2008
- **Type status:** Other material. **Occurrence:** recordedBy: C. Deltshev & M. Komnenov; sex: 1 male, 1 female; **Location:** country: FYR of Macedonia; locality: Galichitsa Mt., Simoncheska Lokva pool; verbatimElevation: 1680 m; **Event:** eventDate: 18-06-2008

##### Distribution

Europeo-Central Asiatic.

##### Notes

First record in Galichitsa Mt.

#### Misumena vatia

(Clerck, 1757)

##### Distribution

Holarctic.

##### Notes

Previously recorded from Ohrid (Drensky 1929, Drensky 1936).

#### Synema globosum

(Fabricius, 1775)

##### Materials

- **Type status:** Other material. **Occurrence:** recordedBy: D. Vidincheva; **Location:** country: FYR of Macedonia; locality: Galichitsa Mt.; verbatimElevation: 600-1800 m; **Event:** eventDate: 26-10-1992
- **Type status:** Other material. **Occurrence:** recordedBy: C. Deltshev & M. Komnenov; sex: 1 male, 2 females; **Location:** country: FYR of Macedonia; locality: Galichitsa Mt., Ohrid, Studenchitsa; verbatimElevation: 690 m; **Event:** eventDate: 18-06-2008
- **Type status:** Other material. **Occurrence:** recordedBy: C. Deltshev & M. Komnenov; sex: 2 females; **Location:** country: FYR of Macedonia; locality: Galichitsa Mt., Dzhafa pool; verbatimElevation: 1650 m; **Event:** eventDate: 17-06-2008
- **Type status:** Other material. **Occurrence:** recordedBy: C. Deltshev & M. Komnenov; sex: 2 females; **Location:** country: FYR of Macedonia; locality: Galichitsa Mt., Stenje vill., Stenjsko Blato bog; verbatimElevation: 850 m; **Event:** eventDate: 17-06-2008

##### Distribution

Palearctic.

##### Notes

Unspecified locality between Resen and Ohrid (Drensky 1929, Drensky 1936, Šilhavý 1944).

#### Oxyptila trux

(Blackwall, 1864)

##### Distribution

Palearctic.

##### Notes

Previously recorded from unspecified locality between Resen and Ohrid (Drensky 1929, Drensky 1936).

#### Thomisus onustus

Walckenaer, 1805

##### Materials

- **Type status:** Other material. **Occurrence:** recordedBy: C. Deltshev & M. Komnenov; sex: 3 males; **Location:** country: FYR of Macedonia; locality: Galichitsa Mt., Stenje vill., Stenjsko Blato bog; verbatimElevation: 850 m; **Event:** eventDate: 17-06-2008
- **Type status:** Other material. **Occurrence:** recordedBy: C. Deltshev & M. Komnenov; sex: 1 male; **Location:** country: FYR of Macedonia; locality: Galichitsa Mt., Tomoros peak; verbatimElevation: 1830 m; **Event:** eventDate: 22-06-2008

##### Distribution

Palearctic.

##### Notes

Previously recorded from Ohrid (Deltshev et al. 2000, Šilhavý 1944).

#### Tmarus piochardi

(Simon, 1866)

##### Distribution

Mediterranean.

##### Notes

Previously recorded from Ohrid, Studenchitsa (Drensky 1929, Drensky 1936).

#### Xysticus acerbus

Thorell, 1872

##### Distribution

Europeo-Central Asiatic.

##### Notes

Previously recorded from unspecified locality between Ohrid and Resen (Drensky 1929, Drensky 1936).

#### Xysticus cristatus

(Clerck, 1757)

##### Distribution

Palearctic.

##### Notes

Previously recorded from Ohrid (Drensky 1929, Drensky 1936).

#### Xysticus erraticus

(Blackwall, 1834)

##### Distribution

European.

##### Notes

Previously recorded from Resen and Ohrid (Drensky 1929, Drensky 1936).

#### Xysticus gallicus

Simon, 1875

##### Materials

- **Type status:** Other material. **Occurrence:** recordedBy: C. Deltshev & M. Komnenov; sex: 1 female; **Location:** country: FYR of Macedonia; locality: Galichitsa Mt., Zhichara; verbatimElevation: 1515 m; **Event:** eventDate: 20-06-2008

##### Distribution

Palearctic.

##### Notes

First record in Galichitsa Mt.

#### Xysticus kempeleni

Thorell, 1872

##### Distribution

Europeo-Central Asiatic.

##### Notes

Previously recorded from Ohrid (Drensky 1929, Drensky 1936).

#### Xysticus kochi

Thorell, 1872

##### Distribution

West Palearctic.

##### Notes

Previously recorded from Resen and Ohrid (Drensky 1929, Drensky 1936).

#### Xysticus lanio

C.L. Koch, 1835

##### Materials

- **Type status:** Other material. **Occurrence:** recordedBy: D. Vidincheva; **Location:** country: FYR of Macedonia; locality: Galichitsa Mt.; verbatimElevation: 600-1800 m; **Event:** eventDate: 26-10-1992

##### Distribution

Palearctic.

##### Notes

First record in Galichitsa Mt.

#### Xysticus macedonicus

Šilhavý, 1944

##### Materials

- **Type status:** Other material. **Occurrence:** recordedBy: C. Deltshev & M. Komnenov; sex: 1 female; **Location:** country: FYR of Macedonia; locality: Galichitsa Mt., Tomoros peak; verbatimElevation: 1830 m; **Event:** eventDate: 22-06-2008

##### Distribution

Balkan endemic.

##### Notes

First record in Galichitsa Mt.

#### Xysticus robustus

(Hahn, 1832)

##### Distribution

Europeo-Central Asiatic.

##### Notes

Previously recorded from Ohrid (Stojićević 1929).

#### Xysticus tenebrosus

Šilhavý, 1944

##### Materials

- **Type status:** Other material. **Occurrence:** recordedBy: C. Deltshev & M. Komnenov; **Location:** country: FYR of Macedonia; locality: Galichitsa Mt., Golem Grad island; verbatimElevation: 842 m; **Event:** eventDate: 20-06-2008

##### Distribution

Northeast Mediterranean.

##### Notes

**Figure 9.**
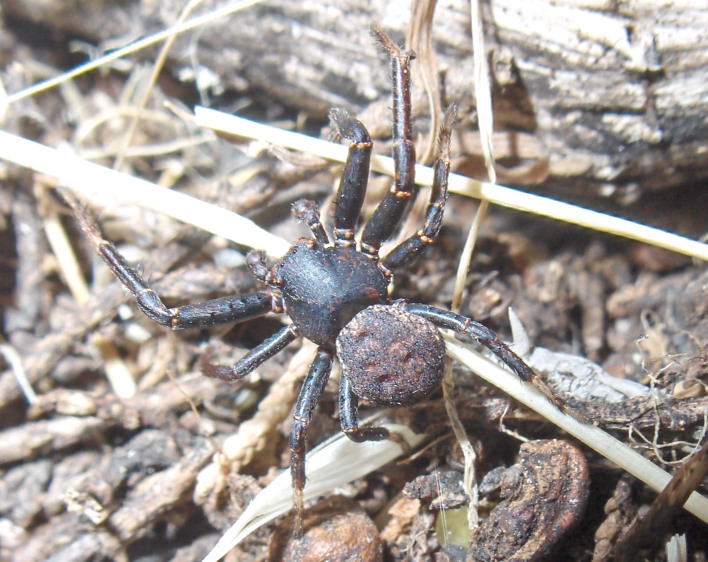
*Xysticus tenebrosus*, typical for the Golem Grad Island.

First record in Galichitsa Mt. (Fig. 9).

#### Xysticus tenebrosus ohridensis

Šilhavý, 1944

##### Distribution

Galichitsa endemic.

##### Notes

Previously recorded from Ohrid (Šilhavý 1944).

#### SALTICIDAE

#### Evarcha arcuata

(Clerck, 1757)

##### Distribution

Palearctic.

##### Notes

Previously recorded from unspecified locality between Ohrid and Prespa Lake (Drensky 1929, Drensky 1936).

#### Evarcha falcata

(Clerck, 1757)

##### Distribution

Palearctic.

##### Notes

Unspecified locality between Resen and Ohrid (Drensky 1929, Drensky 1936).

#### Evarcha laetabunda

(C. L. Koch, 1846)

##### Distribution

Palearctic.

##### Notes

Previosly recorded from Ohrid (Drensky 1929, Drensky 1936).

#### Euophrys frontalis

(Walckenaer, 1802)

##### Materials

- **Type status:** Other material. **Occurrence:** recordedBy: C. Deltshev & M. Komnenov; sex: 1 female; **Location:** country: FYR of Macedonia; locality: Galichitsa Mt., Bugarska Chuka Peak; verbatimElevation: 1797 m; **Event:** eventDate: 19-06-2008

##### Distribution

Palearctic.

##### Notes

First record in Galichitsa Mt.

#### Euophrys rufibarbis

(Simon, 1868)

##### Materials

- **Type status:** Other material. **Occurrence:** recordedBy: B. Petrov; sex: 1 female; **Location:** country: FYR of Macedonia; locality: Galichitsa Mt., Otechevo; verbatimElevation: 1609-1650 m; **Event:** eventDate: 01-05-2008

##### Distribution

Palearctic.

##### Notes

First record in Galichitsa Mt.

#### Heliophanus auratus

C. L. Koch, 1835

##### Materials

- **Type status:** Other material. **Occurrence:** recordedBy: C. Deltshev & M. Komnenov; sex: 1 female; **Location:** country: FYR of Macedonia; locality: Galichitsa Mt., Bugarska Chuka Peak; verbatimElevation: 1797 m; **Event:** eventDate: 19-06-2008

##### Distribution

Palearctic.

##### Notes

First record in Galichitsa Mt.

#### Heliophanus cupreus

(Welckenaer, 1802)

##### Distribution

Palearctic.

##### Notes

Previously recorded from unspecified locality between Resen and Ohrid (Drensky 1929, Drensky 1936).

#### Heliophanus flavipes

(Hahn, 1832)

##### Distribution

Palearctic.

##### Notes

Unspecified locality between Resen and Ohrid (Drensky 1929, Drensky 1936, Stojićević 1929).

#### Heliophanus kochii

Simon, 1868

##### Materials

- **Type status:** Other material. **Occurrence:** recordedBy: C. Deltshev & M. Komnenov; sex: 1 female; **Location:** country: FYR of Macedonia; locality: Galichitsa Mt., Simoncheska Lokva pool; verbatimElevation: 1680 m; **Event:** eventDate: 18-06-2008

##### Distribution

Palearctic.

##### Notes

First record in Galichitsa Mt.

#### Heliophanus lineiventris

Simon, 1868

##### Materials

- **Type status:** Other material. **Occurrence:** recordedBy: C. Deltshev & M. Komnenov; sex: 1 female; **Location:** country: FYR of Macedonia; locality: Galichitsa Mt., Simoncheska Lokva pool; verbatimElevation: 1680 m; **Event:** eventDate: 18-06-2008

##### Distribution

Palearctic.

##### Notes

First record in Galichitsa Mt.

#### Heliophanus melinus

L. Koch, 1867

##### Materials

- **Type status:** Other material. **Occurrence:** recordedBy: C. Deltshev & M. Komnenov; sex: 1 male, 1 female; **Location:** country: FYR of Macedonia; locality: Galichitsa Mt., Zhichara; verbatimElevation: 1515 m; **Event:** eventDate: 20-06-2008

##### Distribution

Palearctic.

##### Notes

First record in Galichitsa Mt.

#### Macaroeris flavicomis

(Simon, 1884)

##### Materials

- **Type status:** Other material. **Occurrence:** recordedBy: C. Deltshev & M. Komnenov; **Location:** country: FYR of Macedonia; locality: Galichitsa Mt., Golem Grad island; verbatimElevation: 842 m; **Event:** eventDate: 20-06-2008

##### Distribution

Northeast Mediterranean.

##### Notes

First record in Galichitsa Mt.

#### Mendoza canestrinii

(Ninni, 1868)

##### Materials

- **Type status:** Other material. **Occurrence:** recordedBy: C. Deltshev & M. Komnenov; sex: 1 male; **Location:** country: FYR of Macedonia; locality: Galichitsa Mt., vill. Stenje, Stenjsko Blato bog; verbatimElevation: 850 m; **Event:** eventDate: 18-06-2008

##### Distribution

Palearctic.

##### Notes

First record in Galichitsa Mt.

#### Menemerus semilimbatus

(Hahn, 1829)

##### Materials

- **Type status:** Other material. **Occurrence:** recordedBy: C. Deltshev & E. Stojkoska; sex: 1 male; **Location:** country: FYR of Macedonia; locality: Galichitsa Mt., Peshtani vill.; verbatimElevation: 719 m; **Event:** eventDate: 31-08-2005

##### Distribution

Mediterranean.

##### Notes

First record in Galichitsa Mt.

#### Myrmarachne formicaria

(De Geer, 1778)

##### Materials

- **Type status:** Other material. **Occurrence:** recordedBy: D. Vidincheva; **Location:** country: FYR of Macedonia; locality: Galichitsa Mt.; verbatimElevation: 600-1800 m; **Event:** eventDate: 26-10-1992

##### Distribution

Palearctic.

##### Notes

First record in Galichitsa Mt.

#### Pellenes moreanus

Metzner, 1999

##### Materials

- **Type status:** Other material. **Occurrence:** recordedBy: C. Deltshev & M. Komnenov; sex: 1 female; **Location:** country: FYR of Macedonia; locality: Galichitsa Mt., Dzhafa pool; verbatimElevation: 1650 m; **Event:** eventDate: 17-06-2008
- **Type status:** Other material. **Occurrence:** recordedBy: C. Deltshev & M. Komnenov; sex: 1 female; **Location:** country: FYR of Macedonia; locality: Galichitsa Mt., Zhichara; verbatimElevation: 1515 m; **Event:** eventDate: 20-06-2008

##### Distribution

Balkan endemic.

##### Notes

First record in FYR of Macedonia.

#### Philaeus chrysops

(Poda, 1761)

##### Materials

- **Type status:** Other material. **Occurrence:** recordedBy: C. Deltshev & M. Komnenov; sex: 2 males, 1 female; **Location:** country: FYR of Macedonia; locality: Galichitsa Mt., Bugarska Chuka Peak; verbatimElevation: 1797 m; **Event:** eventDate: 19-06-2008

##### Distribution

Palearctic.

##### Notes

Previously recorded from Preseka (Lazarov 2004).

#### Phlegra fasciata

(Hahn, 1826)

##### Materials

- **Type status:** Other material. **Occurrence:** recordedBy: C. Deltshev & M. Komnenov; sex: 1 female; **Location:** country: FYR of Macedonia; locality: Galichitsa Mt., Zhichara; verbatimElevation: 1797 m; **Event:** eventDate: 20-06-2008

##### Distribution

Palearctic.

##### Notes

Previously recorded from unspecified locality between Resen – Ohrid (Drensky 1929, Drensky 1936).

#### Pseudeuophrys obsoleta

(Simon, 1868)

##### Materials

- **Type status:** Other material. **Occurrence:** recordedBy: C. Deltshev & M. Komnenov; sex: 1 female; **Location:** country: FYR of Macedonia; locality: Galichitsa Mt., Zhichara; verbatimElevation: 1515 m; **Event:** eventDate: 20-06-2008

##### Distribution

Europeo-Central Asiatic.

##### Notes

First record in Galichitsa Mt.

#### Salticus zebraneus

(C. L. Koch, 1837)

##### Materials

- **Type status:** Other material. **Occurrence:** recordedBy: D. Vidincheva; **Location:** country: FYR of Macedonia; locality: Galichitsa Mt.; verbatimElevation: 600-1800 m; **Event:** eventDate: 26-10-1992

##### Distribution

Palearctic.

##### Notes

Previously recorded from Ohrid (Stojićević 1929).

## Analysis

### Species composition

A total of 294 species of spiders from 31 families have been found in Galichitsa Mt. New for the mountain are 85 species, 20 of them are also new to the spider fauna of FYR of Macedonia. High species richness was recorded for 5 of the families: Linyphiidae – 40 species, Lycosidae – 37 species, Gnaphosidae – 31 species, Araneidae – 27, and Theridiidae – 24 species (Table 2). The degree of exploration of the group in the mountain is about 60%. The number of the species of Galichitsa Mt is high and represents about 45% of the spider fauna of the country (Blagoev 2002). This is also evident from a comparison with the number of spiders reported from the other mountains in the Balkan Peninsula: West Rhodopes – 418 (38%) Deltshev et al. 2011), Pirin – 324 (30%) (Deltshev and Blagoev 1997), Rila – 280 (26%) (Deltshev et al. 2000a), Central Stara Planina – 270 (25%) (Deltshev et al. 2000b).

### Zoogeographical analysis

**Figure 10.**
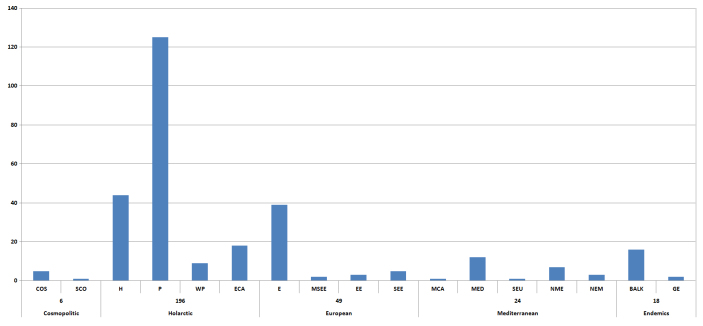
Distribution of the spiders of Galichitsa Mt by zoogeographical categories.

According to their current distribution the established 294 species can be classified into 16 zoogeographic categories, grouped into 5 chorological complexes (Cosmopolitan; Holarctic; European; Mediterranean; Endemic) (Table 3, Fig. 10). The data concerning the general distribution and the chorological characteristics of spiders are taken from Helsdingen (2012), Platnick (2013) and Taglianti et al. (1999).

### Cosmopolitan species complex

Cosmopolitan species complex (COS + SCO) includes only 6 (2%) species widespread in Europe. While the species *Pholcus phalangioides*, *Parasteatoda tepidariorum*, *Steatoda triangulosa*, and *Tegenaria domestica* are connected with human buildings, *Prinerigone vagans* can be accepted as a mountain element. The species is well represented in the woodlands and high altitude zones of the mountains. *Tetragnatha nitens* was reported from the Galichitsa Mt by Drensky (1929), and there is no new data on its distribution in the mountain.

### Holarctic species complex

Holarctic species complex (H + P + WP + ECA) is best represented and comprises 196 (66.7%) species. The Palearctic species s. l. are dominant (125 species, 42.5%), followed by Holarctic (44 species, 15%); Europeo-Central Asiatic (18 species, 6.1%) and West Palearctic (9 species, 3.1%).

This complex includes especially widespread species associated with lowlands, woodlands and high altitude zones of mountains. Most of the species are well represented in the mountains. Characteristic mountain elements are *Frontinelina frutetorum*, *Gongilidium rufipes*, *Pityohyphantes phrygianus*, *Tenuiphantes tenuis*, *Trichoncus affinis*, *Drassodes lapidosus*, *Haplodrassus signifer*. Some xenotopic species are widely distributed in the mountains and reach the highest summits as aeronauts (Thaler 1986). *Meioneta rurestris* and *Oedothorax agrestis*, which inhabit the mountain zone in dense populations, also belong to this complex.

### European species complex

European species complex (E + MSEE + EE + SEE) comprises 49 (16.7%) species. The European species s. l. are dominant (39 species, 13.3%), followed by Southeast European (5 species, 1.7%), Middle and Southeast European (2 species, 0.7%), and East European (3 species, 1.0%).

The complex includes widespread species, which inhabit both lowlands and mountains. Мountain elements are the species *Pardosa albatula*, *Inermocoelotes falciger*, *Inermocoelotes karlinskii*, *Malthonica ferruginea*, *Malthonica silvestris*, *Cybaeus angustiarum*, *Zelotes apricorum*. *Meta menardi* and *Metellina merianae*, found only in caves, can also be placed into this complex.

### Mediterranean species complex

Mediterranean species complex (MCA + M + SE + NM + NEM) includes 24 (8%) species. The Mediterranean species s. l. are dominant (12 species, 4%), followed by North Mediterranean (7 species, 2.4%), North-East Mediterranean and (3 species, 1%) and Mediterranean-Central Asiatic and South European (each by 1 species, 0.3%).

The main part of the established species is presented in the lower and dry parts of the mountain, occurring in the xerothermic oak forests and meadows. Characteristic for the mountain zone are: *Tmarus piochardi*, *Heliophanus kochii*, *Heliophanus melinus*, *Macaroeris flavicomis*, and *Pellenes moreanus*.

### Endemic species complex

Endemic species complex (BP+GA) comprises 18 (6.2%) species. The group of Balkan endemic species (16 species, 5.5%) comprises mainly mountain elements, such as: *Dysdera pectinata*, *Dysderocrates storkani*, *Cheiracanthium macedonicum*, *Zodarion ohridense*, *Zelotes babunaensis*, *Xysticus macedonicus* and *Pellenes moreanus*. They are characteristic for forest and subalpine parts of the mountain, while *Pachygnatha clerckoides* inhabits the coastal vegetation of water basinsin lower parts of the mountain. *Centromerus acutidentatus*, *Lepthyphantes centromeroides*, *Palliduphantes byzantinus*, *Palliduphantes spelaeorum*, *Palliduphantes trnovensis*, *Tegenaria paragamiani* are found only in caves.

The local endemics: *Zora prespaensis* and *Xysticus tenebrosus ohridensis*, can be regarded also as mountain elements. They are known from single localities and need additional faunistic and taxonomic studies.

## Figures and Tables

**Table 1.** List of the localities where spiders have been collected in Galichitsa Mts.

#	Locality	Date	Altitude (in meters)	Published or legator
1	Bugarska Chuka peak	19.6.2008	1801	C. Deltshev & M. Komnenov
2	Crvena Lokva	18.6.2008	1620	C. Deltshev & M. Komnenov
3	Dzhafa pool	18.6.2008	1650	C. Deltshev & M. Komnenov
4	Galichitsa Mt	26.10.1992	600-1800	D. Vidincheva
5	Galichitsa NP		1100-1400	Deeleman-Reinhold and Deeleman 1988
6	Golem Grad island	20.6.2008	860	C. Deltshev & M. Komnenov
7	Lagadin, Ohrid			Deltshev et al. 2000
8	Leskovska Peshtera cave, Leskovec vill (distr. Ohrid)	18.6.2008	1066	C. Deltshev & M. Komnenov
9	Mechkina Dupka cave	20.6.2008	1020	C. Deltshev & M. Komnenov (Šilhavý 1944);
10	Ohrid	30.8.2002	748	Drensky 1929, Drensky 1936; Stojićević 1929; Wunderlich 1973, Wunderlich 1985; G. Blagoev & C. Deltshev
11	Ohrid – Prespa lake			Drensky 1929, Drensky 1936
12	Otechevo		910	Deeleman-Reinhold and Deeleman 1988
13	Peshtani vill. (district Ohrid)	31.8.2005	719	C. Deltshev & E. Stojkoska
14	Petrinska planina (district Resen)			Drensky 1929, Drensky 1936
15	Preseka	30.8.2005	1603	Lazarov 2004; C. Deltshev, G. Blagoev & E. Stojkoska; C. Deltshev & S. Lazarov
16	Resen	30.8.2002	1000	Drensky 1929, Drensky 1936; G. Blagoev & C. Deltshev
17	Resen - Ohrid			Drensky 1929, Drensky 1936; Deeleman-Reinhold & Deeleman
18	Road to Bugarska Chuka peak	19.6.2008	1509	C. Deltshev & M. Komnenov
19	Samatska Peshtera cave	20.6.2008	1436	C. Deltshev & M. Komnenov
20	Simoncheska lokva	18.6.2008	1680	C. Deltshev & M. Komnenov
21	Stenje vill, Stenjsko Blato bog (distr. Ohrid)	17.6.2008	858	C. Deltshev & M. Komnenov
22	Studenchitsa	30.8.2002	690	G. Blagoev & C. Deltshev
23	Sveti Stefan (distr. Ohrid)	31.8.2005	680	C. Deltshev & E. Stojkoska
24	Tomoros peak	22.6.2008	1830	C. Deltshev & M. Komnenov
25	Trapejca vill., cave	9.12.2010	940	C. Deltshev, M. Komnenov & E. Stojkoska
26	Vojla cave	20.6.2008	1508	C. Deltshev & M. Komnenov
27	Zhichara	20.6.2008	1515	C. Deltshev & S. Lazarov; C. Deltshev & M. Komnenov

**Table 2.** Family composition of the spiders of Galichitsa Mt.

	Family	Species number	%
1	Atypidae	1	0.3
2	Scytodidae	1	0.3
3	Pholcidae	4	1.4
4	Segestriidae	2	0.7
5	Dysderidae	5	2
6	Mimetidae	1	0.3
7	Uloboridae	1	0.3
8	Nesticidae	1	0.3
9	Theridiidae	24	8.5
10	Linyphiidae	40	12.7
11	Tetragnathidae	12	4
12	Araneidae	27	9.2
13	Lycosidae	37	12.7
14	Pisauridae	2	0.7
15	Oxyopidae	2	0.7
16	Zoriidae	1	0.3
17	Agelenidae	13	4
18	Cybaeidae	2	0.7
19	Dictynidae	6	2.1
20	Amaurobiidae	2	0.7
21	Titanoecidae	1	0.3
22	Miturgidae	6	2.1
23	Liocranidae	4	1.4
24	Clubionidae	7	2.4
25	Corinidae	5	1.7
26	Zodariidae	3	1
27	Gnaphosidae	31	10.2
28	Sparassidae	1	0.3
29	Philodromidae	13	4.5
30	Thomisidae	19	6.8
31	Salticidae	20	6.8
	**Total:**	**294**	**100%**

**Table 3.** Chorological complexes and zoogeographical categories of Galichitsa Mt Spiders. The chorotypes are abbreviated as follows: COS – Cosmopolitan; SCO – Subcosmopolitan; H – Holarctic; P – Palearctic; WP – West-Palearctic; ECA – European-Central Asiatic; E – European; MSEE – Middle-Southeast European; EE – East European; SEE – Southeast European; MCA – Mediterranean-central Asiatic; MED – Mediterranean; SEU – South European; NME – North Mediterranean; NEM – Northeast Mediterranean; BALK – Balkan endemics; GE – Rhodopean endemics.

COMPLEX	CHOROTYPE		SPECIES	
	Category	Code	Number	%
**Cosmopolitic**	Cosmopolitan	COS	5	1.7
	Subcosmopolitan	SCO	1	0.3
	Total		6	2.0
**Holarctic**	Holarctic	H	44	15
	Palearctic	P	125	42.5
	West Palearctic	WP	9	3.1
	Europeo-Central Asiatic	ECA	18	6.1
	Total		196	66.7
**European**	European	E	39	13.3
	Middle and Southeast European	MSEE	2	0.7
	East European	EE	3	1
	Southeast European	SEE	5	1.7
	Total		49	16.7
**Mediterranean**	Mediterrano-Central Asiatic	MCA	1	0.3
	Mediterranean	MED	12	4
	South European	SEU	1	0.3
	North Mediterranean	NME	7	2.4
	Northeast Mediterranean	NEM	3	1
	Total		24	8
**Endemics**	Balkan endemics	BALK	16	5.5
	Galichitsa endemics	GE	2	0.7
	Total		18	6.2
